# Mitochondrial Lon protease is a gatekeeper for proteins newly imported into the matrix

**DOI:** 10.1038/s42003-021-02498-z

**Published:** 2021-08-16

**Authors:** Yuichi Matsushima, Kazuya Takahashi, Song Yue, Yuki Fujiyoshi, Hideaki Yoshioka, Masamune Aihara, Daiki Setoyama, Takeshi Uchiumi, Satoshi Fukuchi, Dongchon Kang

**Affiliations:** 1 Department of Clinical Chemistry and Laboratory Medicine, Graduate School of Medical Sciences, Kyushu University, 3-1-1 Maidashi, Higashi-ku, Fukuoka, 812-8582 Japan; 2 Department of Life Science and Informatics, Maebashi Institute of Technology, Maebashi, Gunma Japan

**Keywords:** Molecular biology, Biochemistry, Cell biology

## Abstract

Human ATP-dependent Lon protease (LONP1) forms homohexameric, ring-shaped complexes. Depletion of LONP1 causes aggregation of a broad range of proteins in the mitochondrial matrix and decreases the levels of their soluble forms. The ATP hydrolysis activity, but not protease activity, of LONP1 is critical for its chaperone-like anti-aggregation activity. LONP1 forms a complex with the import machinery and an incoming protein, and protein aggregation is linked with matrix protein import. LONP1 also contributes to the degradation of imported, aberrant, unprocessed proteins using its protease activity. Taken together, our results show that LONP1 functions as a gatekeeper for specific proteins imported into the mitochondrial matrix.

## Abstract (web summary)

Yuichi Matsushima et al. revealed that Human ATP-dependent Lon protease (LONP1), a mitochondrial protease with unfolding activity, serves as a gatekeeper for several mitochondrial matrix entering proteins: supporting the folding of required proteins and degrading the aberrant ones.

## Introduction

Mitochondria are essential organelles in eukaryotic cells and involved in many cellular processes, including ATP production. Mitochondria have outer and inner membranes and the space inside the inner membrane is called the matrix. The mitochondrial matrix contains its own genome, mitochondrial DNA (mtDNA); however, mammalian mtDNA encodes only 13 polypeptides involved in oxidative phosphorylation^1,2^. Thus, because the mitochondrial matrix contains nearly 500 polypeptides, almost all the mitochondrial matrix proteins, including the enzymes needed for mtDNA replication, transcription and translation, are synthesized by cytoplasmic ribosomes and imported into mitochondria^3^. Three conserved proteases, Lon, ClpXP and *m-*AAA, are responsible for degrading these imported mitochondrial matrix proteins^4–8^. Lon and ClpXP localize to the mitochondrial matrix, whereas *m-*AAA is anchored to the inner membrane with its catalytic site residing in the matrix.

In humans, the ATP-dependent mitochondrial Lon protease, LONP1, forms a homohexameric ring-shaped structure^7,9^. Human *m-*AAA comprises two subunits, an ATPase family gene 3-like 2 polypeptide (AFG3L2) and paraplegin, and forms homohexameric structures of AFG3L2, or heterohexamers of AFG3L2 and paraplegin^4,5,8^. ClpXP is a barrel-shaped, heterooligomeric complex that is composed of two subunits, the proteolytic subunit, CLPP, and the chaperone subunit, CLPX, which has an ATPase domain^4,5^. LONP1, AFG3L2 and paraplegin each have three domains: an N-terminal domain, a central ATPase domain and a C-terminal protease domain. The N-terminal domain binds the substrate^4,8^. These proteases are members of a superfamily of ATPases associated with diverse cellular activities (AAA + ATPases). For these AAA + proteases, ATPase hydrolysis is used to unfold the substrate protein, and mutations in the ATP binding site of the proteases causes loss of protease activity^4,5,8^. The Lon proteases contain serine protease domains, whereas AFG3L2 and paraplegin belong to the metalloprotease family.

Mutations in the gene encoding LONP1 cause cerebral, ocular, dental, auricular and skeletal anomalies (CODAS) syndrome^10–12^. Cell lines derived from patients show decreases in oxidative phosphorylation activity associated with accumulation of protein aggregates in the mitochondrial matrix^10^. Knockdown of LONP1 also causes mitochondrial respiratory disturbance and protein aggregation in the mitochondrial matrix^13–15^; however, how the impairment of LONP1 causes protein aggregation remains unresolved.

In this study, we investigated how LONP1 prevents protein aggregation. The results suggest that chaperone-like activity, not protease activity, of LONP1 is primarily responsible for its anti-aggregation activity. Moreover, our study strongly suggests that LONP1 binds specific proteins that enter the mitochondria matrix and promotes their solubility.

## Results

### Knockdown of LONP1 increases aggregation of specific proteins in mitochondria

**Fig. 1:**
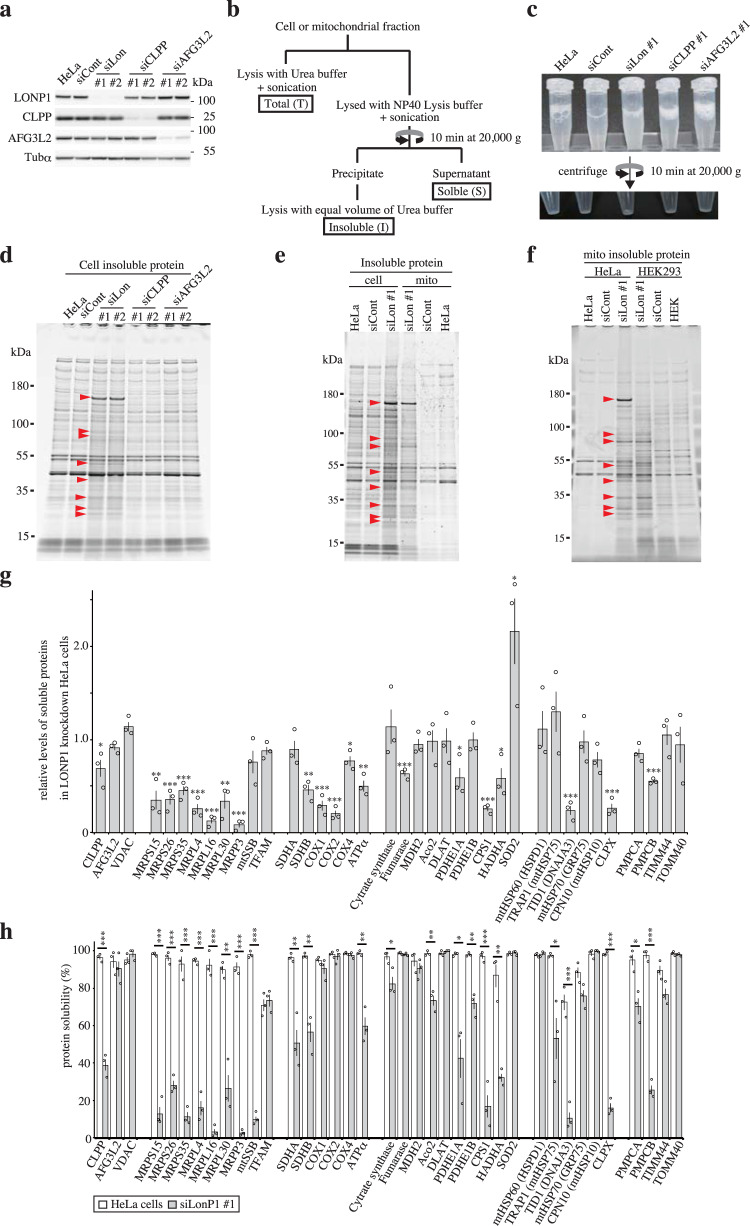
Analysis of aggregated proteins in LONP1 knockdown HeLa cells. Analysis of proteins from non-transfected HeLa cells (HeLa) or HeLa cells treated with siRNA for non-target control (siCont), LONP1 (siLon), CLPP (siCLPP) or AFG3L2 (siAFG3L2). Cells were retransfected 3 d after the first siRNA transfection. Cells were used for experiments 3 d after the second transfection. Protein extracts (10 μg) were separated by SDS-PAGE, transferred to PVDF membranes and probed with antibodies. Molecular weight markers (kDa) are shown to the right of the gel. The experiments were repeated three times and representative data are shown. **a** Immunoblots of mitochondrial matrix proteases in the siRNA-treated HeLa cells. **b** Schematic diagram of the preparation of proteins. **c** The typical preparation of lysates from siRNA-treated HeLa cells. **d** Comparison of insoluble proteins prepared from siRNA-treated HeLa cells. The insoluble proteins were fractionated by 5–20% SDS-PAGE and stained with Oriole Fluorescent Gel Stain. Red arrowheads indicate typical insoluble proteins specific to LONP1 knockdown cells. Molecular weight markers (kDa) are shown to the left of the gel. **e** Comparison of insoluble proteins prepared from cells and mitochondria. **f** Comparison of mitochondrial insoluble proteins prepared from HeLa and HEK293 cells. **g** The relative levels of soluble mitochondrial proteins in LONP1 knockdown HeLa cells determined from the results of the immunoblot analysis using soluble fractions of the knockdown cells (Supplementary Fig. S2). The levels of soluble mitochondrial proteins in LONP1 knockdown cells were normalized against the level of soluble mitochondrial proteins in control cells (*n* = 3). The levels of tubulin-α were used as references. **h** Protein solubility was determined from the results of the immunoblot analysis using soluble and insoluble cell fractions (Supplementary Fig. S2). The protein solubility was quantified as the percentage of soluble proteins in the sum of soluble and insoluble proteins (*n* = 3). Data were analyzed using the Student’s two-tailed *t*-test and are presented as means ± SD (*P < 0.05; **P < 0.01; ***P < 0.001, Student’s *t*-test).

We knocked down LONP1, CLPP and AFG3L2 in HeLa cells using two siRNAs for each protease to characterize the functional effects of the three proteases toward protein aggregation in the mitochondrial matrix (Fig. 1a). The knockdown process was repeated once at 3-d intervals and cells were harvested 6 d after the first transfection. After the cells were lysed by sonication in NP-40 buffer, the lysate from LONP1 knockdown cells was more turbid when compared with that of the lysates from the other knockdown cells (Fig. 1b, c). After centrifugation, the insoluble fractions were treated with an equal volume of urea buffer. SDS-PAGE analysis showed the presence of specific insoluble proteins in LONP1 knockdown cells (Fig. 1d). Results for CLPP or AFG3L2 knockdown cells were almost the same as those for untreated cells. Furthermore, we confirmed that aggregated proteins accumulated in LONP1 knockdown cells (Supplementary Fig. S1a).

Next, we prepared insoluble proteins from the mitochondrial fraction of these knockdown cells (Supplementary Fig. S1b). Most of the LONP1 knockdown-specific insoluble proteins observed in whole cells were found to be mitochondrial (Fig. 1e). Interestingly, many of the specific insoluble proteins from the LONP1 knockdown cells were not major proteins in the total mitochondrial fraction from these cells (Supplementary Fig. S1c). These results indicate that, in LONP1 knockdown cells, specific proteins aggregate in mitochondria. To confirm that mitochondrial localization is critical for protein insolubility, mitochondria-targeted signal-tagged *Discosoma* sp. red fluorescent protein (MtDsRed2) and cytosol-localized *Aequorea coerulescens* green fluorescent protein (AcGFP) were transiently expressed in LONP1 knockdown cells. Most of the MtDsRed2 protein was insoluble in LONP1 knockdown cells, whereas cytosolic AcGFP showed high solubility in both control and knockdown cells (Supplementary Fig. S1d). Similarly, mitochondria-targeted signal-tagged AcGFP (MtAcGFP) was essentially insoluble in LONP1 knockdown cells (Fig. S1e). Moreover, insoluble mitochondrial proteins were observed in LONP1 knockdown HEK293 cells (Supplementary Fig. S1f–h); the insoluble mitochondrial proteins in HeLa and HEK293 cells generally showed identical migration patterns on SDS-PAGE (Fig. 1f and Supplementary Fig. S1i). Next, we analyzed the insoluble proteins from control and LONP1 knockdown cells by mass spectrometry (MS). The insoluble proteins from untreated HeLa cells included a few mitochondrial proteins (Supplementary Fig. S1j). However, there were many mitochondrial proteins among the insoluble proteins from LONP1 knockdown HeLa cells (Supplementary Fig. S1j and Supplementary Data 1). Furthermore, the insoluble mitochondrial proteins from LONP1 knockdown HEK293 cells were almost the same as those from LONP1 knockdown HeLa cells (Supplementary Data 2). Interestingly, in the insoluble mitochondrial proteins from HeLa cells, the top protein at ~170 kDa (indicated by arrowhead) was barely detected in HEK293 cells (Fig. 1f and Supplementary Fig. S1i). MS analysis showed that this protein is Carbamoyl-Phosphate Synthase 1 (CPS1), which is barely expressed in HEK293 cells (Supplementary Fig. S1i and Fig. S1k). Therefore, we presumed that the proteins detected in the insoluble proteins of HeLa and HEK did not completely match because of the difference in the expression level of each protein in mitochondria and the contamination of non-mitochondrial insoluble proteins. These results indicate that most of the aggregated mitochondrial proteins in LONP1 knockdown cells are common between cell types.

### Analysis of mitochondrial protein status in LONP1 knockdown cells

We analyzed the solubility of various mitochondrial proteins in LONP1 knockdown cells (Fig. 1g, h and Supplementary Fig. S2). Many mitochondrial proteins, such as CLPX and a mitochondrial DnaJ co-chaperone (TID1/DNAJA3), were remarkably depleted in the soluble fraction and increased significantly in the insoluble fraction of LONP1 knockdown cells. The levels of other proteins, such as succinate dehydrogenase complex flavoprotein subunit A (SDHA) and mitochondrial heat shock protein 75 kDa (TRAP1/mtHSP75), increased in the insoluble fractions without marked reduction in levels of their soluble form in the knockdown cells. Moreover, some mitochondrial proteins, such as superoxide dismutase 2 (SOD2), mitochondrial transcription factor A (TFAM) and malate dehydrogenase 2 (MDH2), did not accumulate in the insoluble fraction in LONP1 knockdown cells. As expected, mitochondrial outer membrane proteins, such as VDAC and translocase of the outer mitochondrial membrane 40 (TOMM40), were not insoluble in the knockdown cells. Similar results were obtained using HEK293 LONP1 knockdown cells (Supplementary Fig. S3). These results confirm that LONP1 knockdown causes increased insolubility of specific mitochondrial matrix proteins.

### LONP1 and mitochondrial chaperones

**Fig. 2:**
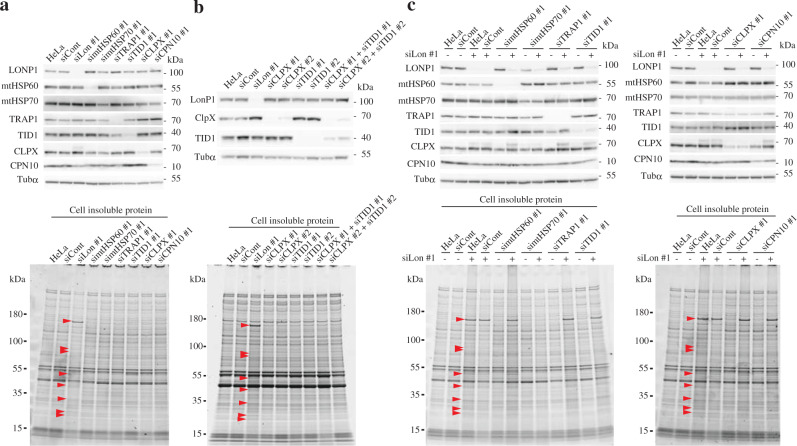
The effects of depletion of mitochondrial chaperone proteins on protein aggregation. **a** Analysis of insoluble proteins from HeLa cells treated with siRNAs. Cells were used for experiments 3 d after the second transfection. *Upper panel*, immunoblot analysis was carried out as described in the legend to Fig. 1a. *Lower panel*, comparison of insoluble proteins prepared from the cells, carried out as described in the legend to Fig. 1d. **b** Analysis of insoluble proteins from HeLa cells treated with siRNAs. *Upper panel*, immunoblot analysis of total proteins from double-knockdown HeLa cells. *Lower panel*, comparison of insoluble proteins prepared from the double-knockdown cells. The experiments were repeated twice and representative data are shown. **c** *Upper panel*, immunoblot analysis of total proteins from double-knockdown HeLa cells. *Lower panel*, comparison of insoluble proteins prepared from the double-knockdown cells. These experiments were repeated twice and representative data are shown.

Chaperone proteins are responsible for protein quality control and loss of chaperone function often causes protein aggregation^16–18^. As described above, in LONP1 knockdown cells, some mitochondrial chaperone proteins, such as TID1 and CLPX, were depleted in the soluble fraction (Fig. 1g, h and Supplementary Fig. S2). To assess whether protein aggregation associated with LONP1 knockdown is due to depletion of functional mitochondrial chaperones, we knocked down each of the mitochondrial chaperone proteins mtHSP70/mortalin/HSPA9, mtHSP60/HSPD1, TRAP1, TID1, CLPX and mitochondrial 10 kDa heat shock protein (CPN10/HSPE1) in HeLa cells using two siRNAs (Fig. 2a and Supplementary Fig. S4a). Analysis of the insoluble proteins from each knockdown cell line showed no significant change in levels of insoluble proteins when compared with that of control cells (Fig. 2a and Supplementary Fig. S4a). Furthermore, since the levels of soluble TID1 and CLPX in LONP1 knockdown cells decreased, we performed a double knockdown of CLPX and TID1. However, in the double knockdown cells, the insoluble proteins specific to LONP1 knockdown were almost undetectable (Fig. 2b). These results suggested that protein aggregation caused by LONP1 knockdown is not primarily due to the depletion of soluble mitochondrial chaperone proteins. In addition, to analyze the synergistic effect of LONP1 and each chaperone on mitochondrial protein quality control, we performed a double knockdown of LONP1 and each mitochondrial chaperone in HeLa cells (Fig. 2c and Supplementary Fig. S4b). The insoluble proteins from mtHSP60/LONP1, TRAP1/LONP1, TID1/LONP1, CLPX/LONP1, or CPN10/LONP1 double-knockdown cells did not significantly change relative to those from LONP1 knockdown cells (Fig. 2c and Supplementary Fig. S4b). These results indicate that these chaperones neither promote nor inhibit the insolubilization of mitochondrial proteins by LONP1 knockdown. However, surprisingly, in the double-knockdown mtHSP70/LONP1, the insoluble proteins specific to LONP1 knockdown were almost undetectable (Fig. 2c and Supplementary Fig. S4b). This result suggests that mtHSP70 is required for accumulation of insoluble proteins associated with LONP1 depletion.

### Mitochondrial protein transport is crucial for protein aggregation caused by LONP1 depletion

**Fig. 3:**
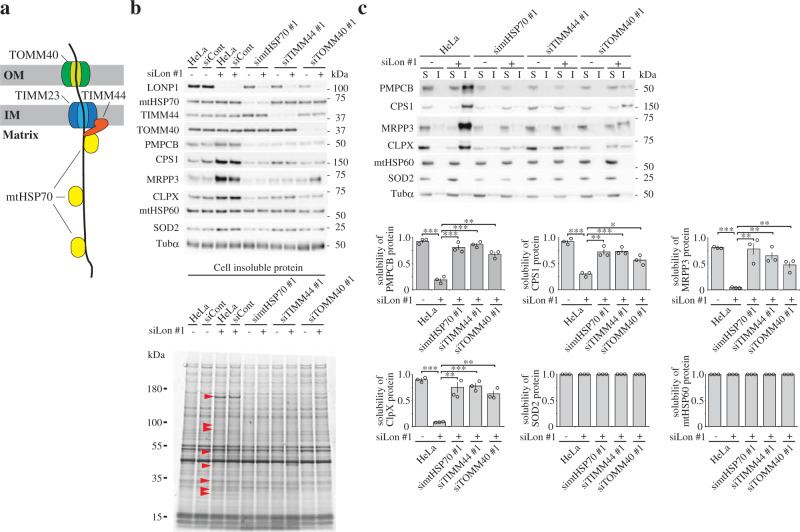
Analysis of the relationship between the mitochondrial protein import machinery and protein aggregation caused by depletion of LONP1. **a** Model of mitochondrial protein import machinery. TOMM40 and TIMM23 are the central pore-forming subunits of the translocase complex in the mitochondrial outer membrane and inner membrane, respectively. TIMM44 is a peripheral inner membrane protein on the matrix side and mediates mtHSP70 attachment to TIMM23, and mtHSP70 binds incoming proteins to facilitate their translocation. **b** Analysis of insoluble proteins from HeLa cells treated with siRNAs. Cells were used for experiments 3 d after the second transfection. *Upper panel*, immunoblot analysis was carried out as described in the legend to Fig. 1a. *Lower panel*, comparison of insoluble proteins prepared from the cells was carried out as described in the legend to Fig. 1d. Red arrowheads indicate typical insoluble proteins specific to LONP1 knockdown cells. The experiments were repeated three times and representative data are shown. **c** *Upper panel*, immunoblot analysis of soluble (S) and insoluble (I) proteins in the knockdown cells was carried out as described in the legend to Supplementary Fig. S2. *Lower panel*, the protein solubility was determined from the results of the immunoblot analysis using soluble and insoluble cell fractions. The protein solubility was quantified as the percentage of soluble proteins in the sum of soluble and insoluble proteins (*n* = 3). Data were analyzed using the Student’s two-tailed *t*-test and are presented as means ± SD (*P < 0.05; **P < 0.01; ***P < 0.001, Student’s *t*-test).

We showed above that mtHSP70 contributes to the accumulation of the insoluble proteins associated with LONP1 depletion. Previous studies showed that mtHSP70 contributes not only to protein folding but also to protein import into the mitochondrial matrix;^19–21^ in the matrix, mtHSP70 binds incoming proteins to facilitate their translocation (Fig. 3a). Thus, we hypothesized that mitochondrial protein import is involved in the accumulation of insoluble proteins associated with LONP1 depletion. To test this hypothesis, we performed double-knockdown of LONP1 and an additional component of the mitochondrial protein import machinery, translocase of the inner mitochondrial membrane 44 (TIMM44) or TOMM40, in HeLa cells^19–21^. TOMM40 is the central pore-forming subunit of the translocase complex in the mitochondrial outer membrane, and TIMM44 is a peripheral inner membrane protein located on the matrix side and mediates mtHSP70 attachment to the pore-forming translocase subunit of the inner mitochondrial membrane 23 (TIMM23) (Fig. 3a). Similar to mtHSP70/LONP1 double-knockdown cells, levels of the insoluble proteins specific to LONP1 knockdown dramatically decreased in TIMM44/LONP1 and TOMM40/LONP1 double-knockdown cells (Fig. 3b and Supplementary Fig. S5a). Moreover, we analyzed the solubility of carbamoyl-phosphate synthase 1 (CPS1), PMPCB, MRPP3 and CLPX, all proteins that are insoluble when LONP1 is depleted. Immunoblot analysis showed that in these double-knockdown cells, the solubility of all these proteins was partially, or almost fully, recovered to the levels found in control cells (Fig. 3c and Supplementary Fig. S5b). These results suggest that protein transport into the mitochondrial matrix is a critical step for accumulation of insoluble proteins associated with LONP1 depletion.

### Protease activity of LONP1 is not critical for protein aggregation

**Fig. 4:**
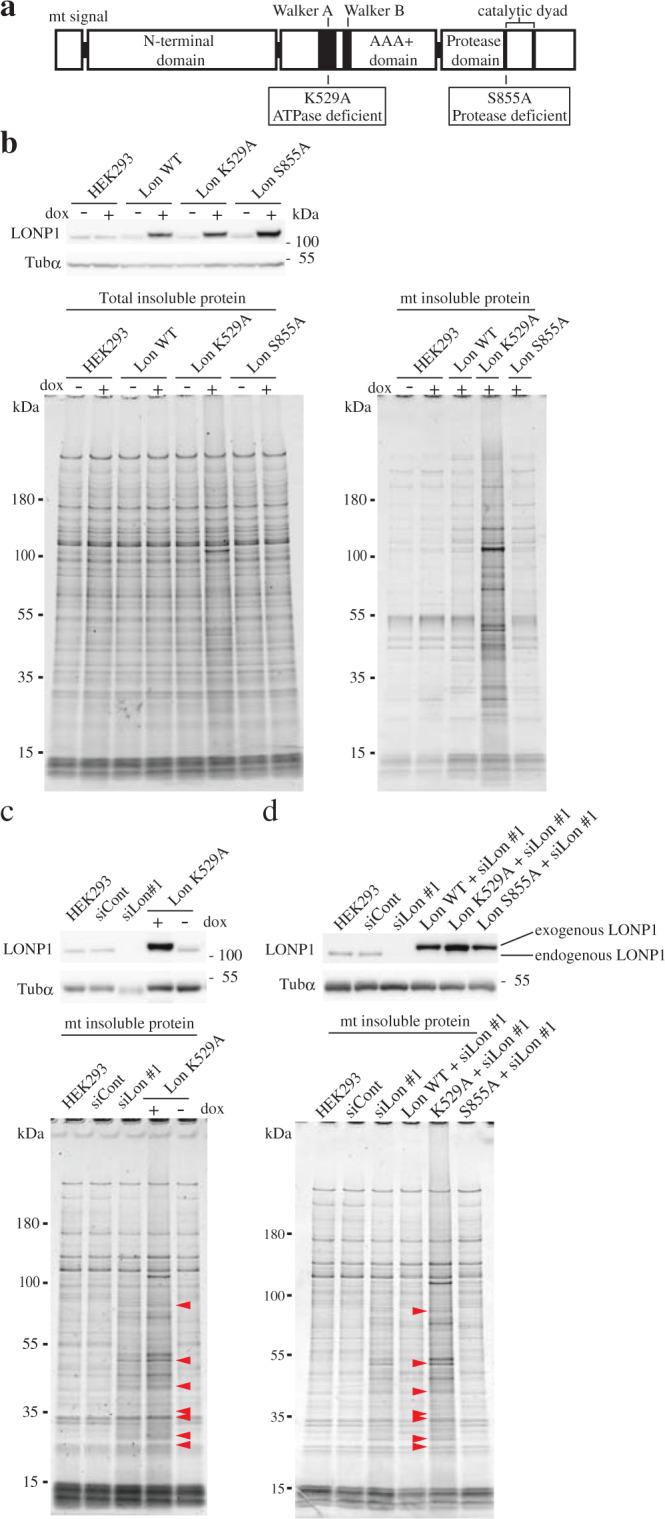
Expression of LONP1 active-site variants in HEK293 cells. **a** Domain architecture of the Lon protease: an N-terminal domain, a central AAA + ATPase domain and a C-terminal protease domain. The AAA + domain consists of two subdomains, Walker A and Walker B. The C-terminal protease domain contains a serine and lysine dyad in the active site. We constructed doxycycline-inducible plasmids expressing 3× FLAG-tagged wild**-**type LONP1 and variants carrying either a K529A or S855A exchange. K529 in the Walker A motif is essential for ATP hydrolysis whereas S855 is part of the serine protease motif in the active site. **b** HEK293 cell lines carrying no vector (HEK293) or pcDNA5/FRT/TO-3×FLAG-LONP1 (Lon WT, L529A, or S855A) were cultured for 6 d in the presence or absence of 1 ng/mL doxycycline. *Upper panel*, immunoblot analysis of total proteins from the cell lines was carried out as described in the legend to Fig. 1a. *Lower panel*, comparison of insoluble mitochondrial proteins prepared from the cell lines was carried out as described in the legend to Fig. 1d. Comparison of insoluble mitochondrial proteins prepared from the induced cell lines. **c** Comparison of the insoluble mitochondrial proteins from HEK293 cells treated with siRNAs, and HEK293 cell line expressing ATPase-deficient LONP1. *Upper panel*, immunoblot analysis of total proteins from the cells. *Lower panel*, comparison of insoluble mitochondrial proteins prepared from the cell lines. Red arrowheads indicate typical insoluble proteins specific to LONP1 knockdown cells. **d** Comparison of the insoluble mitochondrial proteins from HEK293 cells treated with siRNAs, and endogenous LONP1-depleted HEK293 cells expressing exogenous LONP1 and its variants. *Upper panel*, immunoblot analysis of total proteins from the cells. *Lower panel*, comparison of insoluble mitochondrial proteins prepared from the cell lines. These experiments were repeated twice and representative data are shown.

To assess whether the protease activity of LONP1 is critical for protein insolubilization, we established stable HEK293 cell lines expressing the doxycycline (Dox)-inducible codon-optimized Flag-tagged wild-type LONP1 protease, and mutants carrying either a K529A or S855A substitution (Fig. 4a and Supplementary Fig. S6a). S855 is located in the active site of the serine protease family, and K529 is a conserved residue in the Walker A motif. Previous in vitro analysis showed that S855 is essential for protease activity, but not for ATP hydrolysis activity, and K529 is necessary for both ATP hydrolysis and protease activity^22^. Since studies showed that TFAM, which is the most abundant mtDNA structural factor, is specifically degraded by LONP1 associated with mtDNA depletion^23,24^, we investigated the degradation of TFAM associated with mtDNA depletion in cells expressing LONP1 mutants. The cell lines expressing LONP1 K529A or S855A showed retardation of TFAM degradation following ethidium bromide treatment (which causes mtDNA depletion in cells) (Supplementary Fig. S6c). These results indicate that both LONP1 mutants exhibit a dominant negative phenotype, which is caused by the formation of mixed oligomeric forms, composed of the mutants and endogenous LONP1. After 6 d of induction in the presence of Dox, the cell line expressing LONP1 K529A accumulated insoluble proteins in mitochondria identical to that observed for LONP1 knockdown cells (Fig. 4b, c and Supplementary Fig. S7a). Similar results were obtained using HEK293 cell line expressing Walker B mutant LONP1, E591A (Supplementary Fig. S7b). Surprisingly, in the cell line expressing S855A, the insoluble mitochondrial proteins did not change when compared with that of the control cells (Fig. 4b, c and Supplementary Fig. S7a). To exclude the possibility that endogenous LONP1 affects mitochondrial protein aggregation in cells expressing LONP1 S855A, we selectively knocked down endogenous LONP1 in HEK293 cells expressing the LONP1 mutants (Supplementary Fig. S6b). The knockdown process was repeated twice at 3-d intervals and the cells were harvested 6 d after the first transfection. Even when endogenous LONP1 was depleted, the specific insoluble proteins associated with LONP1 knockdown were scarcely detectable in cells expressing the LONP1 S855A mutant (Fig. 4d). These results indicate that loss of the protease function in LONP1 is not critical for the accumulation of specific insoluble proteins, and that the LONP1 S885A mutant retains activity that prevents protein aggregation associated with endogenous LONP1 depletion. In contrast, the cell line expressing LONP1 K529A exhibited accumulation of the specific insoluble proteins associated with LONP1 knockdown, with or without endogenous LONP1 knockdown. These results reveal that the ATPase activity of LONP1 is essential for the solubilization of the specific proteins. Since the ATPase activity is essential for chaperone-like activity in the yeast Lon protease^25^, chaperone-like activity of LONP1 toward proteins newly imported into the matrix may be important for suppression of aggregation.

### LONP1 maintains the soluble state of newly imported proteins

**Fig. 5:**
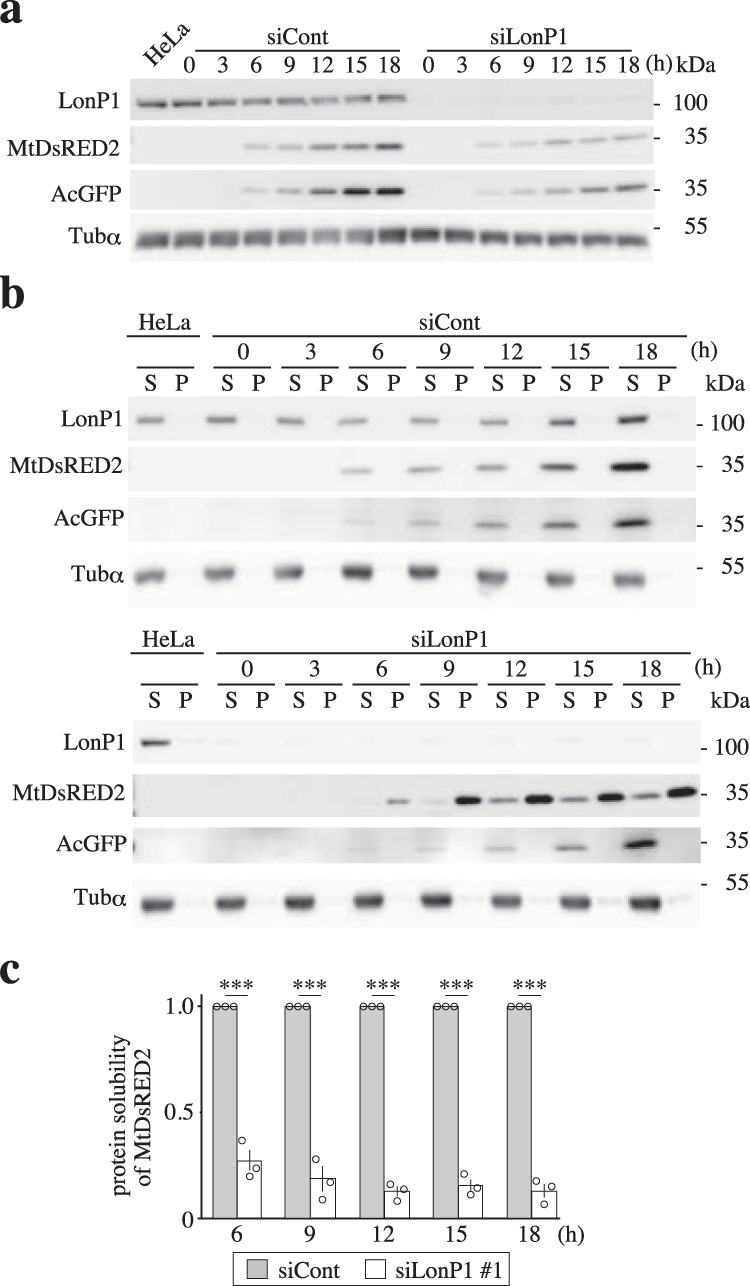
LONP1 helps solubilize newly imported proteins. Immunoblot analysis of transiently expressed mitochondria-targeted Mt-DsRed2 and cytosol-targeted AcGFP in LONP1-depleted cells. HeLa cells were retransfected 3 d after the first siRNA transfection. Transfection of the pDsRed2-Mito and pAcGFP vectors was performed 2 d after the second siRNA transfection. Cells were collected every 3 h following plasmid transfection. Total, soluble (S) and insoluble (I) proteins from cells were prepared as described in the Methods and in Fig. 1b. **a** Immunoblot analysis of total proteins from the cells. **b** Immunoblot analysis of soluble (S) and insoluble (I) proteins from the cells. **c** The protein solubility of Mt-DsRed2 was quantified as the percentage of soluble proteins in the sum of soluble and insoluble proteins (*n* = 3).

To assess whether LONP1 helps to solubilize newly imported proteins, we performed time course analysis of transient expression of mitochondrially-targeted Mt-DsRed2 in LONP1 knockdown cells. Two days after the second siRNA transfection, the pDsRed2-Mito and pAcGFP vectors were transfected into the cells and then the cells were collected every 3 h. The majority of Mt-DsRed2 had already aggregated at the same time point as it appeared 6 h after transfection (Fig. 5a–c). In contrast, newly imported Mt-DsRed2 is not insoluble in the control cells and the cytosolic AcGFP showed high solubility in both control and knockdown cells (Fig. 5a, b). We have also confirmed that the half-life of mtDsREd2 exceeds 6 h (Supplementary Fig. S8). These results show that most of the newly imported Mt-DsRed2 had immediately aggregated in LONP1 knockdown cells.

### LONP1 interacts with the mitochondrial protein import machinery

**Fig. 6:**
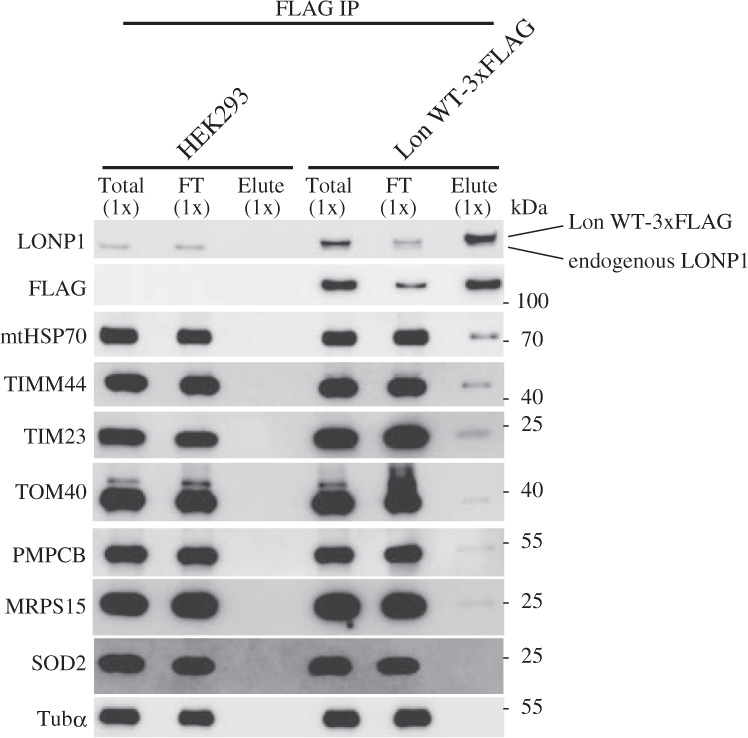
LONP1 forms a complex with the mitochondrial protein import machinery. HEK293 cells carrying inducible plasmids expressing 3× FLAG-tagged wild**-**type LONP1 were cultured for 3 d in the presence of 1 ng/mL doxycycline. The cells were extracted with RIPA buffer after treatment with the reversible crosslinker (DSP). The supernatant was recovered as the total protein (Total) following sonication and centrifugation. The FLAG-tagged LONP1 protein was immunoprecipitated using anti-FLAG-tag mAb-magnetic beads. After magnetic separation, unbound proteins were recovered as the flow-through (FT). The magnetic beads were incubated twice with RIPA buffer containing FLAG peptides and recovered as eluate (Elute). After cleavage of a disulfide bond in DSP, all samples were analyzed by immunoblotting. The experiments were repeated three times and representative data are shown.

The above-presented results indicate that proteins that are insoluble because of LONP1 knockdown may be substrates for the chaperone-like function of LONP1, and that protein transport into the mitochondrial matrix is a critical step for the solubilization of proteins. To assess whether LONP1 cooperates with the mitochondrial protein import machinery, we performed coimmunoprecipitation analysis using stably expressing FLAG-tagged LONP1. Immunoblot analysis of the complexes recovered with an anti-FLAG-tag mAb showed putative substrates MRPS16 and PMPCB in the LONP1-FLAG complex, whereas SOD2, which is not insoluble in LONP1 knockdown cells, was not detected in the complex (Fig. 6). Physical interaction of LONP1 and the mitochondrial protein import machinery proteins mtHSP70, TIMM44, TIMM23 and TOMM40 was also confirmed in cells expressing FLAG-tagged LONP1 (Fig. 6). Moreover, immunoprecipitation analysis with anti-TIMM23 also showed that LONP1 interacts with components of the mitochondrial protein import machinery (Supplementary Fig. S9a). Since TOMM40 and TIMM23 are core pore-forming components in the mitochondrial inner and outer membrane, respectively, the interaction between these machinery components and LONP1 may be indirect through incoming proteins that are associated with LONP1 on the matrix side. Consistent with this concept, the association of LONP1 with the mitochondrial protein import machinery was absent when cells were treated with carbonyl-cyanide m-chlorophenyl hydrazone (CCCP), an OXPHOS uncoupler, which blocks one of the earliest import steps (Supplementary Fig. S9b). Similarly, depletion of TIMM23 disrupted the association of LONP1 with the import machinery (Supplementary Fig. S9c). Furthermore, LONP1 did not interact with the TIMM22 complex^26^, which mediates the import of membrane proteins into the mitochondrial inner membrane (Supplementary Fig. S9d). Taken together, these results imply that LONP1 contributes to the solubilization of specific mitochondrial proteins by its chaperone-like activity at the stage of mitochondrial matrix protein import.

### LONP1 plays dual roles: a protease and a chaperone for newly imported proteins

**Fig. 7:**
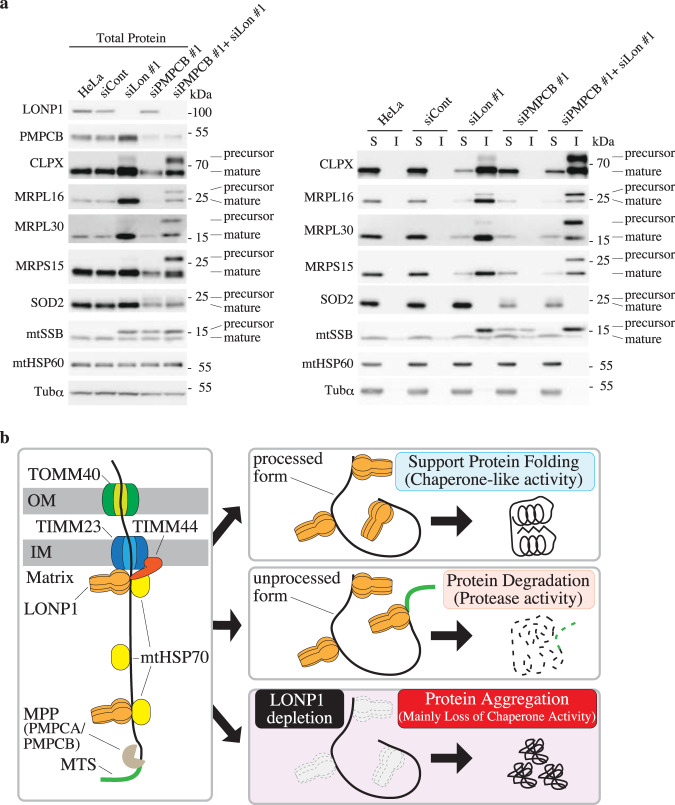
LONP1 specifically degrades some unprocessed mitochondrial proteins. **a** Analysis of proteins from HeLa cells treated with siRNAs. The cells were used for experiments 3 d after the second transfection, *Left panel*, immunoblot analysis of total proteins from cells was carried out as described in the legend to Fig. 1a. *Right panel*, immunoblot analysis of soluble (S) and insoluble (I) proteins in the knockdown cells was carried out as described in the legend to Supplementary Fig. S2. **b** Model of LONP1 activity toward imported proteins. *Left panel*, LONP1 associates with incoming proteins. *Right panel*, LONP1 assists folding and/or assembly of the imported proteins using its chaperone-like activity. Unprocessed imported proteins are degraded by LONP1. Imported proteins aggregate in the mitochondrial matrix of LONP1 knockdown cells or LONP1 dominant negative cells.

Interestingly, immunoblotting results for the mitochondrial single-strand binding protein (mtSSB) showed an additional slower migrating signal in LONP1 knockdown cells, in addition to the normal migration signal (Supplementary Fig. S2). Because the soluble protease subunit of mitochondrial processing peptidase (PMPCB) is depleted in LONP1 knockdown cells (Fig. 1g, h and Supplementary Fig. S2), the additional slower migrating signal was estimated to be the unprocessed form of mtSSB. To confirm this, we knocked down PMPCB or another subunit of the mitochondrial processing peptidase, PMPCA, in HeLa cells. The mobility of the mtSSB precursor in PMPCA or PMPCB knockdown cells was the same as that of the additional mtSSB signal in LONP1 knockdown cells (Supplementary Fig. S10a), indicating that the additional slower migrating signal of mtSSB is the unprocessed form of mtSSB. In PMPCB or PMPCA knockdown cells, the levels of some mitochondrial proteins such as CLPX, MRPS15, MRPL16, MPRL30 and SOD2 decreased without an increase of their corresponding precursor proteins (Supplementary Fig. S10a). We then hypothesized that LONP1 selectively degrades the precursor form of the proteins. To test this hypothesis, we double-knocked down LONP1 and PMPCB in HeLa cells. The levels of the precursor forms of CLPX, MRPL30, MRPL16 and MRPS16 were found to increase dramatically in PMPCB/LONP1 double-knockdown cells (Fig. 7a). Nearly all of these precursor proteins were insoluble in the double-knockdown cells. These results indicate that LONP1 specifically degrades these unprocessed proteins, whereas the mature form is solubilized by LONP1. Different to these proteins, the mtSSB precursor protein was detected in cells depleted of LONP1, PMPCB, or PMPCB and LONP1. Although the mtSSB precursor protein was partially soluble in PMPCB knockdown cells, almost all of the mtSSB precursor protein was insoluble in LONP1 or LONP1/PMPCB knockdown cells (Fig. 7a). This suggests that LONP1 contributes to the solubilization of the precursor mtSSB protein via its chaperone-like activity. In contrast, some precursor mtSSB proteins might also be substrates for the protease activity of LONP1 since the amount of precursor mtSSB protein usually decreases in PMPCB knockdown cells when compared with that of LONP1 knockdown cells (Fig. 7a and Supplementary Fig. S10a). The level of mature SOD2 decreased, whereas the level of precursor SOD2 increased slightly in PMPCB knockdown cells; however, no change was observed for PMPCB/LONP1 double-knockdown cells relative to PMPCB knockdown cells. This indicated that, like other proteins, precursor SOD2 is relatively unstable, but the degradation of this precursor form is not specifically performed by LONP1. The above-presented results indicate that LONP1 associates with specific incoming proteins. To test whether LONP1 interacts with the specific incoming mitochondrial protein import machinery, we performed coimmunoprecipitation analysis using an in vitro import assay with the mitochondrial precursor proteins, MRPL30-3xFLAG and SOD2-3xFLAG (Supplementary Fig. S10b). MRPL30 was used as a representative of proteins that tended to be insoluble in LONP1 knockdown cells, and SOD2 was used as a representative of proteins whose solubility was not affected by knockdown of LONP1. Immunoblot analysis with an anti-LONP1 antibody showed both the unprocessed and mature forms of MRPL30-3xFLAG in the complex, whereas both of the SOD2-3xFLAG forms were not detected in the complex. These results indicate that LONP1 interacts with specific incoming mitochondrial proteins. Moreover, we confirmed that imported MRPL30-3xFLAG was partially insoluble in LONP1 knockdown mitochondria, whereas SOD2-3xFLAG is soluble. (Supplementary Fig. S10c). Taken together, these results suggest that LONP1 selectively acts as a protease or chaperone on specific newly imported proteins.

## Discussion

We found that depletion of LONP1 causes accumulation of aggregated mitochondrial proteins, whereas accumulation of the aggregated proteins was not detected in cells knocked down for CLPP or AFG3L2 (Fig. 1d and Supplementary Fig. S1b). These results suggest that LONP1 has a specific anti-aggregation role in the mitochondrial matrix, and that other matrix proteases, CLPP and *m-*AAA, do not complement the anti-aggregation effects of LONP1. The aggregated proteins from LONP1-depleted HeLa cells were almost identical to the aggregated proteins from HEK293 cells expressing ATPase-deficient LONP1, or LONP1 knockdown HEK293 cells (Figs. 1f and 4c, Supplementary Data 2). Moreover, most of the aggregated proteins are not abundant proteins found in mitochondria (Supplementary Fig. S1c). These results strongly indicate that very specific proteins aggregate because of LONP1 deficiency and most of these aggregated proteins are common to different cell lines. Mitochondrial chaperone proteins, such as mtHSP70 and TID1, have been reported to exhibit protein folding and anti-aggregation activity in the matrix space^16–18^. In LONP1 knockdown cells, the solubility of some mitochondrial chaperone proteins, TID1 and CLPX, was significantly reduced; however, the specific aggregated proteins were not detected in TID1/CLPX double-knockdown cells (Figs. 1g, h and 3b). Similarly, the depletion of other mitochondrial chaperones did not cause a significant increase in the aggregation of specific proteins (Fig. 2a and Supplementary Fig. S4a). These results indicate that aggregation induced by LONP1 knockdown was not primarily caused by the depletion of those functional mitochondrial chaperones and that they are not functionally redundant with LONP1 in anti-aggregation activity. Taken together, our data showed that LONP1 has inherent anti-aggregation effects in the mitochondrial matrix.

Unexpectedly, we could not detect a significant increase of specific insoluble proteins in cells expressing protease-deficient LONP1, whereas a marked accumulation of aggregated proteins was detected in cells expressing ATPase-deficient LONP1 (Fig. 4). These results strongly suggest that the protease activity of LONP1 is not critical for anti-aggregation activity. As observed for LONP1 depletion, expressing ATPase-deficient LONP1 reduced specific mitochondrial proteins in the soluble fraction (Supplementary Fig. S7a). If the accumulation of the insoluble proteins were simply caused by a deficiency in LONP1 protease activity that degrades misfolded or damaged proteins, the aggregated proteins would accumulate without a significant decrease of the soluble proteins. The peroxisomal Lon protease, a homolog of LONP1, has folding activity toward chemically denatured proteins^26^. Moreover, wild-type and proteolytically inactive yeast Lon promotes the assembly of mitochondrial complexes I and V. Thus, it has been proposed that Lon may function as an ATP-dependent molecular chaperone^25^. While Bezawork-Geleta et al. Report that LONP1 degrades chemically denatured proteins in vitro, so it is possible that LONP1 contributes to protein solubilization in cooperation with other factors^27^. Here, we showed a chaperone-like activity that promotes the solubilization of specific mitochondrial matrix proteins. These previous findings support our concept that LONP1 has unfolding activity to enable proteolysis and chaperone-like activity for maintenance of native conformations of specific proteins. Hereafter we describe the latter activity of LONP1 as chaperone-like activity to functionally distinguish this function from the proteolytic activity of LONP1.

Although LONP1 specifically degrades TFAM^23,24^, the protein solubility of TFAM is not affected in LONP1-depleted cells. This indicates that the substrates for the protease activity of LONP1 are not identical to the substrates for the chaperone-like activity of LONP1. More surprisingly, the accumulation of the aggregated proteins was dramatically reduced when protein import into the mitochondrial matrix was suppressed by the co-knockdown of LONP1 and mtHSP70, TIMM44, or TOMM40 (Fig. 3b, c and Supplementary Fig. S5a, b). Indeed, in these co-knockdown cells, the solubility levels of the specific matrix proteins were almost the same as those in control cells (Fig. 3c). These results indicate that aggregation is dependent on protein import into the matrix or newly imported proteins aggregate. LONP1 coprecipitated with substrates for its chaperone-like activity. In addition, LONP1 was physically associated with proteins involved in mitochondrial matrix protein import such as mtHSP70, TIMM44, TIMM23 and TOMM40, which have also been listed in the MS analysis of LONP1-associated proteins (Fig. 6)^13,28^. These results indicate that LONP1 forms a complex with the mitochondrial protein import machinery and each specific imported protein.

In LONP1 knockdown cells, some matrix side peripheral membrane proteins in the respiratory chain aggregated, such as SDHA and SDHB, whereas integral membrane proteins such as cytochrome c oxidase subunit I (COX1), COX2, COX4, or AFG3L2 barely aggregated (Fig. 1g, h and Supplementary Fig. S2). Similarly, MS analysis showed that many peripheral membrane proteins of the mitochondrial respiratory complexes aggregate in LONP1 knockdown cells (Supplementary Data 1). Unlike the peripheral membrane proteins, the integral inner membrane proteins are not imported via the TIMM44–mtHSP70 complex^21^, which is associated with LONP1 (Fig. 3). Thus, our data suggest that LONP1 primarily exhibits chaperone-like function toward newly imported matrix-localized proteins that are transported via the TIMM44-mtHSP70 complex and not toward integral inner membrane proteins. Because LONP1 knockdown resulted in the accumulation of aggregated proteins in the mitochondrial matrix, it appears that LONP1 does not affect mitochondrial matrix protein import per se. These findings are consistent with the result that an interaction between LONP1 and the mitochondrial inner membrane translocase, TIMM22, was not detected (Supplementary Fig. S9d). Taken together, our study suggests that LONP1 binds the mitochondrial protein import complex and helps to solubilize specifically newly imported proteins *via* its chaperone-like activity.

Our list of insoluble proteins in LONP1 knockdown cells is partially different from previous reports. For example, unlike our results, COX2 was included in the insoluble fraction in previous reports^10,13^. This may stem from methodology differences. While our insoluble protein isolation method used NP-40 as the detergent, previously reported methods used Triton X-100 or n-dodecyl-d-maltoside as the detergent. Thus, we compared these three preparation methods using LONP1 knockdown cells (Supplementary Fig. S11a). Interestingly, there was no difference in the distribution of ClpX and Tid1 in the soluble/insoluble fraction among these three methods. However, unlike in our method, COX2 was more abundant in the insoluble fraction in the previously reported methods. Unlike the previously reported methods^10,13^, our method includes a sonication step after solubilization with the detergent, indicating that insoluble COX2 in the knockdown cells may have been solubilized by the sonication. To confirm this, a sonication step was added after dissolution of Triton X-100 or n-dodecyl-D-maltoside. In each case, it was confirmed that insoluble COX2 was significantly reduced by ultrasonic treatment. (Supplementary Fig. S11b). These results indicate that the insoluble state of COX2 was different from the insoluble state of ClpX and TID1 and that our method selectively isolates extremely insoluble proteins caused by LONP1 depletion. Additionally, a decrease in COX2 expression was observed in LONP1 knockdown cells (Supplementary Fig. S11).

Interestingly, we found that mitochondrial ribosomal proteins, CPS1, CLPX, MRPP3 and TID1, which form complexes or oligomers, selectively aggregate in LONP1 knockdown cells (Fig. 1g, h and Supplementary Fig. S2). This indicates that LONP1 supports the assembly of some protein complexes. However, pyruvate dehydrogenase complex component E2 (DLAT), mtHSP60, CPN10 and MDH2 also form multimers, but did not aggregate in LONP1 knockdown cells (Fig. 1g, h and Supplementary Fig. S2). Although the pyruvate dehydrogenase E1 component subunit alpha (PDHE1A) and beta (PDHE1B) constitute pyruvate dehydrogenase as a tetramer^29^, PDEH1A is insoluble, whereas PDHE1B is predominantly soluble in LONP1 knockdown cells (Fig. 1g, h and Supplementary Fig. S2). A similar phenomenon was observed for mitochondrial processing peptidase comprising PMPCA and PMPCB (Fig. 1g, h and Supplementary Fig. S2)^5,8^. Intriguingly, overexpression of the Walker A or B mutant of LONP1, which functions in a hexamer, insolubilized LONP1 itself (Supplementary Fig. S7b). It has been reported in in vitro experiments that the walker A and walker B mutants of LONP1 or Lon protease form multimers, but their ATPase activity and protease activity are lost^22,30,31^. Our results consistent with these reports (Supplementary Fig. S7b). Moreover, the insoluble LONP1 (105 kDa) was confirmed in the protein staining results of Walker A mutant-expressing cells (Fig. 4a, b). This suggests that the chaperone-like activity of LONP1 contributes to the solubilization of LONP1 itself.

As described above, it is unknown how proteins that aggregate in LONP1 knockdown conditions are selected. We observed many mitochondrial ribosomal proteins in the aggregates formed (Fig. 1h). Many ribosomal complex proteins have been classified as intrinsically disordered proteins. Intrinsically disordered proteins are proteins that contain non-folded or unstructured regions and a recent report showed that the susceptibility of proteins to aggregate positively correlates with the presence of intrinsically disordered regions^32^. To investigate the involvement of intrinsically disordered regions, we used a prediction tool, SPOT-Disorder2, and compared the ratio of the intrinsically disordered regions and the protein solubility in LONP1 knockdown cells (Fig. 1h)^33^. Interestingly, proteins that show low-solubility in LONP1 knockdown cells have higher proportions of intrinsically disordered regions (Supplementary Fig. S12). Accordingly, LONP1 may preferentially support solubilization of proteins that have a high proportion of intrinsically disordered regions. Clearly, more detailed physiological and biochemical studies are required to understand the mechanism of protein aggregation caused by LONP1 deficiency.

Because cleavage of pre-sequences enables proper folding and functioning of mature proteins, many unprocessed proteins adopt incorrect structures. LONP1 may recognize the folding state of newly imported proteins and degrade misfolded or unfolded immature proteins. Bezawork-Geleta et al. show that LONP1 degrades the partially deleted folding-incompetent form ornithine transcarbamylase in vitro^27^. Very recently, Rendón et al. reported an increase in the level of insoluble unprocessed proteins in endogenous LONP1 depleted cells expressing exogenous protease-deficient LONP1^13^. These insoluble unprocessed proteins may be protease substrates for LONP1. We found that LONP1 is essential not only for the solubilization of imported proteins, but also for the degradation of abnormal imported proteins, such as unprocessed proteins that retain their signal peptide (Fig. 7a). This indicates that LONP1 selectively uses its protease or chaperone-like function according to the state of the imported protein. Although LONP1 is a protease, our results suggest that it also has chaperone-like activity. How LONP1 correctly uses its protease activity and chaperone-like activity is currently unclear. Structural analysis has shown that the substrate recognized by LONP1 is guided to the protease chamber and degraded. One possibility is that the mechanism of substrate recognition for the chaperone activity differs from that for the protease activity. For example, the substrate may bind to the outside of the barrel of LONP1 rather than to the inside of the barrel structure, to exhibit chaperone-like activity. Further detailed analysis is needed to resolve these questions.

From these studies, our current notion is that LONP1 functions as a gatekeeper for specific or a certain set of newly imported mitochondrial matrix proteins (Fig. 7b). First, LONP1 associates with incoming specific proteins. Next, it assists protein folding and/or assembly using its chaperone-like activity. If specific newly imported proteins are unprocessed, these proteins fail to fold properly and are degraded by the protease activity of LONP1. In contrast, these proteins are not degraded and subsequently aggregate in LONP1 deficient cells. However, since most aggregated proteins are processed, the chaperone-like activity of LONP1 would function to circumvent aggregation effects. A recent report showed that the depletion of PMPCB causes an increase in some unprocessed proteins in LONP1 knockdown fibroblast cells^13^. Our results are consistent with this report. Moreover, we also showed that accumulation of most of the unprocessed proteins in LONP1 knockdown cells is due to the loss of protease activity of LONP1 and not to the depletion of PMPCB (Fig. 7a).

In general, dysfunction of the mitochondrial respiratory chain causes mitochondrial diseases that affect tissues with high-energy demands such as brain, skeletal muscle, kidney, heart and liver. Some autosomal recessive mutations in *LONP1* cause typical mitochondrial diseases^30,34^. However, most autosomal recessive mutations in *LONP1* are associated with the CODAS syndrome, which has broader symptoms than typical mitochondrial diseases^10,11^. In our study, the depletion of LONP1 caused the aggregation of a broad range of mitochondrial proteins that contribute not only to mitochondrial respiration but also to extensive functions such as mitochondrial metabolism, and protein processing, stability and folding (i.e., chaperone functions) (Fig. 1g, h, Supplementary Fig. S2 and Supplementary Data 1). These diverse effects may be one explanation as to why a *LONP1* mutation causes the CODAS syndrome. A very recent paper reported that two patients with a homozygous missense *LONP1* mutation showed pyruvate dehydrogenase deficiency^30^. Because soluble PDHE1A depletion is associated with its aggregation in LONP1 knockdown cells, the decrease in functional PDHE1A by the *LONP1* mutation may be a cause of this disease.

In conclusion, our study suggests that LONP1 functions as a gatekeeper for specific proteins imported into the mitochondrial matrix. LONP1 supports folding of incoming proteins using its chaperone-like function, and degrades abnormal imported proteins by combining its protease and unfolding activities.

## Methods

### Cell lines and cell culture

HeLa cells (ATCC) were maintained in DMEM (4.5 g/L glucose) with L-Gln and sodium pyruvate (Nacalai Tesque, 08458-45), supplemented with 10% fetal bovine serum (FBS) and uridine (50 mg/L). The Flp-In™ T-REx™ 293 Cell Line (HEK293) was procured from Thermo Fisher Scientific and maintained in DMEM (4.5 g/L glucose) with L-Gln and sodium pyruvate (Nacalai Tesque, 08458-45), supplemented with 10% FBS, uridine (50 mg/L), blasticidin (15 mg/mL) and zeocin (100 mg/mL) at 37 °C under 5% CO_2_. Stable cell lines were generated by cotransfecting pOG44 with pCDNA5/FRT/TO vectors encoding the gene for codon-modified 3× FLAG-tagged LONP1 or its variants in a 5:1 ratio using Lipofectamine LTX with the Plus™ Reagent (Thermo Fisher Scientific) according to the manufacturer’s instructions. After 2–4 weeks of selection using blasticidin (10 mg/mL) and hygromycin B (150 mg/mL), viable colonies were expanded. Protein expression was induced with doxycycline (1 ng/mL).

### siRNA transfection

For the first siRNA transfection, cells were plated at 30% confluence in 10-cm dishes and transfected 24 h later using the Lipofectamine RNAiMax Reagent (Thermo Fisher Scientific) according to the manufacturer’s instructions. After 2 d, the cells were plated at 30–40% confluence in 10-cm dishes and retransfected 24 h later. Three days after the second siRNA, cells were harvested by trypsinization. The siRNAs are listed in Supplementary Data 3.

### Ethidium bromide treatment

HEK293 cells with expression vectors for LONP1 and its variants were cultured in the presence of 1 ng/mL doxycycline for 3 d. The medium was then replaced with new medium containing 150 ng/mL ethidium bromide and 1 ng/mL doxycycline, and cells were cultured for a further 3 d. Total cell extracts were analyzed by immunoblotting. Data were analyzed using the Student’s two-tailed *t*-test and are presented as the mean ± SD.

### Preparation of insoluble proteins from cells/mitochondria

Cells were washed twice with PBS and divided into two tubes. Cells were lysed with urea lysis buffer (8 M urea, 10 mM Tris-HCl [pH 7.5], 1% SDS and 10 mM EDTA) to obtain total protein samples (T). After sonication (5 s, eight times), the protein concentration was measured with a Protein Assay Bicinchoninate Kit (Nacalai Tesque). For the preparation of insoluble protein samples, cells were lysed with NP-40 lysis buffer (1% NP-40, 5 mM Tris-HCl [pH 8.0], 150 mM NaCl and protease inhibitor cocktail without EDTA) on ice. After sonication (5 s, eight times, on ice), the protein concentration was measured with the Protein Assay Bicinchoninate Kit, and diluted to 2 mg/mL with the NP-40 lysis buffer. Lysates (800 µL) were centrifuged (20,000 *g* for 10 min, 4 °C) to yield the supernatant and pellet fractions. The supernatant was recovered as the soluble fraction (S). The pellet was washed twice with PBS and treated with an equal volume (800 µL) of urea lysis buffer. For complete dissolution of the pellet, the mixture was vigorously stirred for about 1 h. The resultant solution was used as the insoluble fraction (I). Such protein extracts were fractionated by 2–20% SDS-PAGE, and stained with Oriole Fluorescent Gel Stain (Bio-Rad). For MS analysis, the insoluble fractions were treated with 8 M urea in 0.5 M Tris-HCl (pH 8.0). Isolation of mitochondria from cells was performed using an EzSubcell Fraction (ATTO) subcellular fractionation kit, according to the manufacturer’s instructions. The protein concentration of the mitochondrial fraction was measured with a Protein Assay Bicinchoninate Kit (Nacalai Tesque). For the preparation of mitochondrial fractions, the mitochondrial protein concentration in the NP-40 lysis buffer was diluted to 1 mg/mL with the NP-40 buffer and 200 µL of the lysate was used for further preparation.

In Fig. S11a, the LONP1 knockdown cells were prepared with two different methods described in previous studies^10,13^. (DDM) Cells were extracted with extraction buffer containing 1.5% n-dodecyl-d-maltoside and protease inhibitor cocktail without EDTA for 30 min on ice and the lysate was centrifuged at 20,000 *g* and 4 °C for 20 min. The supernatant was collected as the soluble fraction (S). The pellet was lysed with urea lysis buffer and the solution was used as the insoluble fraction (I). (TX100) Cells were lysed in Triton X-100 buffer (50 mM Tris, pH 7.5, 300 mM NaCl, and 0.5% Triton X-100) supplemented with protease inhibitor cocktail without EDTA. The cells were incubated for 15 min on ice, and then centrifuged at 14,000 r.p.m. for 15 min at 4 °C. The supernatant was collected as the soluble fraction (S). The pellet was lysed with urea lysis buffer and the solution was used as the insoluble fraction (I). In Fig. S11b, A sonication step (5 s, eight times, on ice) was added after the extraction with n-dodecyl-D-maltoside or Triton X-100 containing buffers.

### Anti-LONP1 antibody preparation

To express the C-terminal region of LONP1 in *Escherichia coli*, a PCR fragment of *LONP1* cDNA encoding amino acid residues Ala639 to Arg959 was cloned into pGEX-4T-1 (GE healthcare) cleaved with *Bam*HI and *Sal*I. Bacterial cells harboring the plasmid were grown in LB medium containing ampicillin and expression was induced with isopropyl-β-D-thiogalactopyranoside. The glutathione S-transferase fusion protein was purified by using Glutathione Sepharose 4B according to the manufacturer’s instructions (GE healthcare). Rabbits were immunized by injection of the fusion protein in Freund’s adjuvant.

### Immunoblotting

Total protein samples (10 μg) were resolved on 5–20% or 10-20% SDS-polyacrylamide gels (Wako) and subsequently transferred to polyvinylidene fluoride membranes (Bio-Rad) by semidry transfer using a Trans-Blot Turbo (Bio-Rad). Membranes were preincubated for 1 h with Block One (Nacalai Tesque), followed by incubation overnight with Can Get Signal solution 1 (Toyobo) containing primary antibodies. Membranes were washed four times with phosphate-buffered saline (PBS) containing 0.1% Tween 20, incubated for 1.5 h with Can Get Signal solution 2 (Toyobo) containing horseradish peroxidase-conjugated anti-rabbit or mouse IgG (GE Healthcare), and washed with PBS containing 0.1% Tween 20. Protein bands were visualized using Western Lightning ECL Pro or Ultra (PerkinElmer). ECL luminescence was quantified on an ImageQuant LAS 4000 (GE Healthcare). The intensity of immunoblotting signals was quantified using ImageQuant software (GE Healthcare). The ratios between the target proteins and loading control (tubulin-α) were calculated and normalized. Data were analyzed using the Student’s two-tailed *t*-test and are presented as the mean ± SD. The list of antibodies used in these experiments and their dilution rates are listed in Supplementary Data 3. The soluble and insoluble proteins obtained from 10 μg of total protein extract were loaded on the gel.

### Protein solubility ratio and relative levels of soluble proteins

Protein solubility was determined from the results of immunoblot analysis using soluble and insoluble fractions. Specifically, protein solubility was quantified as the percentage of soluble proteins to the sum of soluble and insoluble proteins. Data were analyzed using the Student’s two-tailed *t*-test and are presented as the mean ± SD. The relative levels of soluble mitochondrial proteins were determined from the results of the immunoblot analysis using the soluble fractions of the cells. The levels of soluble mitochondrial proteins in LONP1 knockdown cells were normalized against the level of soluble mitochondrial proteins in the control cells (*n* = 3). The levels of α-tubulin were used as internal control.

### Analysis of mitochondrially-targeted DsRed2/AcGFP solubility

HeLa cells were cultured in six-well dishes. Transfection of siRNAs was performed using the Lipofectamine RNAiMAX Reagent (Thermo Fisher Scientific) according to the manufacturer’s instructions. Cells were retransfected 3 d after the first siRNA transfection. A day after the second siRNA transfection, cotransfection of pDsRed2-Mito Vector (Clontech) with pAcGFP-N1 (Clontech) in a 1:1 ratio was performed using Lipofectamine LTX with the Plus Reagent according to the manufacturer’s instructions. The cells were cultured for two more days. Total cell extracts were analyzed by immunoblotting, as described above. The experiments were repeated three times. For the analysis of mitochondrially-targeted AcGFP solubility, in the place of the cotransfection, the pAcGFP-Mito Vector (Clontech) was used for the transfection. Data were analyzed using the Student’s two-tailed *t*-test and are presented as the mean ± SD.

### Vector construction

Doxycycline-inducible LONP1 vectors, pcDNA5/FRT/TO-3xFLAG-LONP1 (Lon WT), pcDNA5/FRT/TO-3xFLAG-LONP1 (Lon K529A) and pcDNA5/FRT/TO-3xFLAG-LONP1 (Lon S855A) were constructed as follows. Synthesized codon-changed 3× FLAG-tagged LONP1 cDNA (Fig. S6a) (Thermo Fisher Scientific) was introduced into the doxycycline-inducible vector pcDNA5/FRT/TO (Thermo Fisher Scientific). Expression vectors carrying mutated LONP1 were prepared by QuikChange mutagenesis with *Pfu* Turbo DNA polymerase (Agilent). A specific primer pair was used for each mutant, as listed in Supplementary Data 3.

### Coimmunoprecipitation analysis

HEK293 cells with pcDNA5/FRT/TO vectors encoding 3× FLAG-tagged LONP1 were cultured for 3 d in the presence of 1 ng/mL doxycycline. The cells (6 × 10^6^) were washed three times in PBS, then suspended in PBS containing 2 mM dithiobis(succinimidylpropionate) (DSP) and incubated on ice for 30 min. The reaction was quenched with glycine (to a final concentration of 20 mM). The cells were washed twice in PBS and extracted in 800 µL RIPA buffer (Cell Signaling) containing a protease inhibitor cocktail without EDTA and sonicated eight times for 5 s each, on ice. The extract was cleared by centrifugation at 12,000 × *g* (4 °C, 10 min), and the supernatant (Total) was recovered and an aliquot of total cell lysate was subjected to immunoblot analysis. The cell lysate (500 µL) was mixed with 30 µL anti-FLAG-tag mAb-magnetic beads (MBL, M185-11) and immunoprecipitation was performed by rotating the tubes for 90 min at 4 °C. After magnetic separation, the supernatant was recovered as flow-through (FT) and the beads were washed four times with RIPA buffer. FLAG peptides (25 mg/mL) in RIPA buffer (250 µL) were added and the mixture was rotated for 20 min at 4 °C. The eluate (Elute) was collected using a magnetic rack and the elution process was repeated once. To cleave the disulfide bond in DSP, SDS sample buffer containing 50 mM dithiothreitol (DTT) was added to each fraction. For detection of the immunoprecipitated proteins in immunoblotting, TidyBlot (Bio-Rad) and horseradish peroxidase-conjugated anti-rabbit/mouse IgG (GE Healthcare) were used. Coimmunoprecipitation analysis with anti-TIMM22, anti-TIMM23 and anti-LONP1 antibodies (Proteintech) was performed using Dynabeads™ Protein G (Thermo Fisher Scientific) according to the manufacturer’s directions.

### Aggresome assay

Analysis of aggregated proteins was performed with the PROTEOSTAT® protein aggregation assay (Enzo) according to the manufacturer’s instructions.

### LC–MS/MS analysis

For MS analysis, the insoluble fractions from HeLa cells were treated with 8 M urea in 0.5 M Tris-HCl (pH 8.0). Urea-treated lysates containing 100 µg of protein were reduced with DTT (final 5 mg/mL) at 37 °C for 30 min, subjected to carbamidomethylation of cysteine using iodoacetamide (final 8 mg/mL) at 37 °C for 30 min, and then diluted with four volumes of 50 mM ammonium bicarbonate. The protein solution was subjected to trypsin digestion using MonoSpin Trypsin (GL Sciences) and the digests were purified using MonoSpin C18 (GL Sciences) according to the manufacturer’s protocols. The methanol eluate was evaporated and then dissolved with 0.1% formic acid containing 2% acetonitrile. About 1 µg of peptide was separated with an Easy-nLC1000 system (Thermo Fisher Scientific) using an Acclaim PepMap100 trap column (20 × 0.075 mm, 3 µm; Thermo Fisher Scientific) and an Acclaim PepMap RSCL analytical column (150 × 0.05 mm, 2 µm; Thermo Fisher Scientific), and analyzed on a Q-Exactive Orbitrap mass analyzer (Thermo Fisher Scientific). Data analysis was performed using Proteome Discoverer software (Thermo Fisher Scientific) for protein identification through the SequestHT algorithm against the human protein UniProt database. Identified proteins (score > 10) are listed in Supplementary Data 1.

### Analysis of disordered regions in the proteins

The intrinsically disordered regions were analyzed by SPOT-Disorder2 (http://sparks-lab.org/jack/server/SPOT-Disorder2/)^33^. The protein sequences, those mitochondrial targeting signals are removed, were used for the analysis. The ratios of the intrinsically disordered regions in mature proteins are plotted in Supplementary Fig. S12.

### Mitochondrial import assay

The in vitro mitochondrial import assay was performed according to the method of Mackenzie et al. with minor modifications^35^. Specifically, cell-free translation of human recombinant MRPL30-3xFLAG and SOD2-3xFLAG proteins was performed using the TnT Quick Coupled Transcription/Translation System (Promega), according to the manufacturer’s instructions. The PCR-generated DNA templates containing the T7 promoter were amplified with the primers shown in Supplementary Data 3. The total reaction volume was adjusted to 50 μL with nuclease-free water. Reactions were incubated for 90 min at 30 °C, and then 250 mM sucrose was added. Mitochondria were isolated from HeLa cells with EzSubcell fraction kit (ATTO) and suspended to a final concentration of 1 mg/mL in Import buffer (20 mM HEPES-KOH, pH 7.5, 5 mM MgOAc2, 80 mM KOAc and 250 mM sucrose) containing 2 mM ATP, 0.4 mM ADP and 1 mM dithiothreitol. Next, 40 μL of the recombinant protein solution was mixed with 110 μL of the mitochondrial solution and incubated at 30 °C for 20, 40 and 60 min with intermittent gentle mixing. For coimmunoprecipitation analysis, mitochondria incubated for 20 min were used. Mitochondria incubated for 20 min were also used for immunoprecipitation experiments. Mitochondria were re-isolated by centrifugation, suspended in PBS containing the crosslinker, dithiobis(succinimidyl propionate) (DSP), and incubated on ice for 30 min. The reaction was quenched with glycine (at a final concentration of 20 mM), washed twice with Import buffer, extracted with 800 μl of RIPA buffer containing a protease inhibitor cocktail without EDTA and sonicated for 8 × 5 s on ice. The extract was cleared by centrifugation at 12,000 *g* (4 °C, 10 min), the supernatant (Total) was collected and an aliquot of the total lysate was subjected to immunoblot analysis. Coimmunoprecipitation analysis was performed as described above. Mitochondria incubated for 60 min were used for isolation of soluble and insoluble proteins. Mitochondria were collected by centrifugation and incubated with trypsin (100 μg/mL) om ice for 30 min in sucrose buffer (20 mM HEPES-KOH, pH 7.5, 1 mM EDTA and 250 mM sucrose). After washing the mitochondria with sucrose buffer containing a protease inhibitor cocktail, mitochondria were lysed with NP-40 buffer to prepare soluble and insoluble proteins as described above.

### Statistics and reproducibility

Statistical analyses were performed using JMP Pro 14.2 (JMP, Tokyo, Japan) and Microsoft Excel for Mac (Microsoft). For the statistical analyses, at least three independent experiments were performed. Student’s *t*-test was used to evaluate comparisons between two individual groups. *P*-value < 0.05 were considered statistically significant.

### Reporting summary

Further information on research design is available in the Nature Research Reporting Summary linked to this article.

## Supplementary information

Supplementary Information

Description of Additional Supplementary Files

Supplementary Data 1

Supplementary Data 2

Supplementary Data 3

Supplementary Data 4

Reporting Summary

## Data Availability

The data that support the findings of this study are available from the corresponding author (YM) upon reasonable request. Source data (Supplementary Data 4) are provided with this paper. The mass spectrometry proteomics data are available from ProteomeXchange and jPOST. The accession numbers for the aggregated proteins from HeLa LONP1 knockdown cells are PXD027377 for ProteomeXchange and JPST001258 for jPOST, respectively. The accession numbers for the aggregated proteins from HEK293 LONP1 knockdown cells are PXD027376 for ProteomeXchange and JPST001259 for jPOST, respectively.
