# Supplementary material for: Mitochondrial Lon protease is a gatekeeper for proteins newly imported into the matrix
Source: Commun Biol. 2021 Aug 16;4:974. doi: 10.1038/s42003-021-02498-z (PMC8368198; doi:10.1038/s42003-021-02498-z)

Supplementary Information

Title: Mitochondrial Lon protease is a gatekeeper for proteins newly imported into the matrix

Running title: LONP1 maintains the soluble state of newly imported proteins

Yuichi Matsushima[1,*], Kazuya Takahashi[1], Song Yue[1], Yuki Fujiyoshi[1], Hideaki Yoshioka[1], Masamune Aihara[1], Daiki Setoyama[1], Takeshi Uchiumi[1], Satoshi Fukuchi[2] and Dongchon Kang [1,*]

[1]Department of Clinical Chemistry and Laboratory Medicine, Graduate School of Medical Sciences, Kyushu University, 3-1-1 Maidashi, Higashi-ku, Fukuoka 812-8582, Japan;

[2]Department of Life Science and Information, Maebashi Institute of Technology, Maebashi, Gunma 371-0816, Japan

*Correspondence: Yuichi Matsushima, Tel: +81 92 642 5752; Fax: +81 92 642 5772; Email: matsush5@cclm.med.kyushu-u.ac.jp or Dongchon Kang, Tel: +81 92 642 5748; Fax: +81 92 642 5772; Email: kang@cclm.med.kyushu-u.ac.j

Supplementary Fig. S1 Depletion of LONP1 results in accumulation of aggregated proteins in mitochondria. a The levels of aggregated proteins in HeLa cells treated with siRNAs. Aggregated proteins were analyzed with the PROTEOSTAT® protein aggregation assay kit. **b** Comparison of insoluble mitochondrial proteins from siRNA-treated HeLa cells. Red arrowheads indicate typical insoluble proteins specific to LONP1 knockdown cells. **c** Comparison of total (T), soluble (S) and insoluble (I) mitochondrial proteins from HeLa treated with siRNAs. Each lane was loaded with total (T), soluble (S) or insoluble (I) proteins obtained from 10 μg of total protein extract. **d, e** Immunoblot analysis of transiently expressed mitochondrially/cytosol-targeted DsRed2/AcGFP in LONP1 depleted cells. Transfection of the vectors was performed 1 d after the second siRNA transfection. After 2 more days, total, soluble (S) and insoluble (I) proteins from the cells were prepared and immunoblot analysis was carried out. The experiments were repeated three times. The protein solubility is quantified as the percentage of soluble protein to the sum of soluble and insoluble proteins. Data were analyzed using the Student's two-tailed *t*-test and are presented as means ± SD (****P* < 0.001, Student's *t*-test). **f** Immunoblot analysis of total

proteins from HEK293 cells treated with siRNAs. The experiments were repeated three times and representative data are shown. **g** Comparison of insoluble proteins prepared from siRNA-treated HEK293 cells. **h** Comparison of insoluble mitochondrial proteins from siRNA-treated HEK293 cells. **i** Comparison of total (T) and insoluble (I) mitochondrial proteins from HeLa or HEK293 cells treated with siRNA for LONP1. **j** Numbers of identified proteins from MS analysis of the insoluble fraction of HeLa cells (HeLa) or HeLa cells treated with siRNA for LONP1 (siLon #1). The numbers of identified proteins (score >10) from mitochondria and other compartments are shown in black and gray, respectively. The detailed aggregated mitochondrial protein list is shown in Supplementary Data 1. **k** Immunoblot analysis of total proteins from HeLa and HEK293 cells.

Supplementary Fig. S2 Immunoblot analysis of total, soluble and insoluble proteins in LONP1 knockdown HeLa cells. Analysis of proteins from HeLa cells treated with siRNAs. Cells were retransfected 3 d after the first siRNA transfection. Cells were used for experiments 3 d after the second transfection. Total, soluble (S) and insoluble (I) proteins

from the cells were prepared and immunoblot analysis was carried out. Each lane was loaded with total (T), soluble (S) or insoluble (I) proteins obtained from 10 μg of total protein extract. The experiments were repeated three times and representative data are shown. Statistical analysis is shown in Fig. 1g, h.

Supplementary Fig. S3 Immunoblot analysis of total, soluble and insoluble proteins in LONP1 knockdown HEK293 cells. Analysis of proteins from HEK293 cells treated with siRNAs. Cells were retransfected 3 d after the first siRNA transfection. Cells were used for experiments 3 d after the second transfection. Total, soluble (S) and insoluble (I) proteins from cells were prepared and immunoblot analysis was carried out. Each lane was loaded with total (T), soluble (S) or insoluble (I) proteins obtained from 10 μg of total protein extract. The experiments were repeated three times and representative data are shown.

Supplementary Fig. S4 The effects of the depletion of mitochondrial chaperone proteins on protein aggregation using another set of siRNAs. Analysis of insoluble

proteins from HeLa cells treated with siRNAs. The experiments were repeated twice and representative data are shown. **a** *Upper panel*, cells were retransfected 3 d after the first siRNA transfection and immunoblot analysis was carried out. Each lane was loaded with 10 μg total (T) protein extract. *Lower panel*, analysis of insoluble proteins from the siRNA-treated cells. Comparison of insoluble proteins prepared from the cells was carried out as described in the legend to Fig. 1d. **b** *Upper panel*, immunoblot analysis of total proteins from double-knockdown HeLa cells, *Lower panel*, analysis of insoluble proteins from the double-knockdown HeLa cells.

Supplementary Fig. S5 Analysis of the relationship between the mitochondrial protein import machinery and protein aggregation caused by LONP1 depletion using another set of siRNAs. Analysis of insoluble proteins from HeLa cells treated with siRNAs. Cells were retransfected 3d after the first siRNA transfection. The experiments were repeated two times and representative data are shown. **a** *Upper panel*, immunoblot analysis was carried out as described in the legend to Fig. 1a. *Lower panel*, comparison of insoluble proteins

prepared from the cells was carried out as described in the legend to Fig. 1d. **b** Soluble (S) and insoluble (I) proteins from cells were prepared and immunoblot analysis was carried out. Each lane was loaded with soluble or insoluble proteins obtained from 10 μg of total protein extract.

Supplementary Fig. S6 Expression of LONP1 active-site variants in HEK293 cells. a cDNA sequences of 3× FLAG-tagged codon-changed LONP1 cDNA. The start and stop codons are highlighted in yellow. The sequence for the 3× FLAG-tag is highlighted in orange. The sequence complementary to the siLon #1 target sequence is highlighted in yellow. **b** Comparison of the sequences of the siLon #1 target and the codon-changed cDNA region. Mismatches are indicated with asterisks. **c** Dynamics of TFAM proteins during mtDNA depletion in HEK293 cells overexpressing LONP1 and its variants. HEK293 cell lines having the vectors encoding Lon WT, Lon K529A, or Lon S855A, were cultured for 3 d in the presence of 1 ng/mL doxycycline and cultured for 3 more days in the presence of 150 ng/mL ethidium bromide and 1 ng/mL doxycycline. Cells were harvested

before and after ethidium bromide treatment at 0, 1 and 3 d. *Left panel*, immunoblot analysis was carried out as described in the legend to Fig. 1a. *Right panel*, relative TFAM/tubulin-α ratio. The experiments were repeated three times. Data were analyzed using the Student's two-tailed *t*-test and are presented as the mean ± SD (*$P < 0.05$; **$P < 0.01$, Student's *t*-test).

Supplementary Fig. S7 Immunoblot analysis of total, soluble and insoluble proteins in HEK293 cells expressing LONP1 and its variants. a HEK293 cells containing the vector encoding Lon WT, Lon K529A or Lon S855A were cultured for 6 d in the presence of 1 ng/mL doxycycline. Total, soluble (S) and insoluble (I) proteins from the cells were prepared and immunoblot analysis was performed. Each lane was loaded with total, soluble or insoluble proteins obtained from 10 μg of total protein extract. The experiments were repeated three times and representative data are shown. **b** HEK293 cells containing the vector encoding Lon WT, Lon K529A or Lon E591A were cultured for 6 d in the presence of 1 ng/mL doxycycline. Total, soluble (S) and insoluble (I) proteins from the cells were

prepared and immunoblot analysis was performed. The experiments were repeated twice and representative data are shown.

Supplementary Fig. S8 Cycloheximide chase assay of the MtDsRed2 protein. *Upper panel*, HeLa cells were transiently transfected with pDsRed2-Mito. Fifteen hours after the transfection, cells were cultured in the presence of 200 μg/mL emetine and 100 μg/mL cycloheximide. The cells were isolated at the indicated times. Total cell extracts (10 μg) were analyzed by immunoblot analysis as described in the legend to Fig. 1. The results are representative of three independent experiments. *Lower panel*, the levels of MtDsRed2 were standardized to the levels of tubulin-α. The experiments were repeated three times.

Supplementary Fig. S9 The mitochondrial protein import machinery forms a complex with LONP1. a HEK293 cells were treated with the reversible crosslinker, DSP, and protein was extracted with RIPA buffer. After sonication and centrifugation, the

supernatant was collected as the total protein (Total). TIMM23 protein was immunoprecipitated using the anti-TIMM23 antibody and protein G magnetic beads. After magnetic separation, unbound proteins were collected as the flow-through (FT). The beads were incubated with urea buffer and the elution was collected (Elute). All samples were analyzed by immunoblotting after cleavage of the disulfide bond in DSP. The experiments were repeated three times and representative data are shown. **b** HEK293 cells harboring the vector encoding Lon WT, Lon K529A or Lon S855A were cultured for 3 d in the presence of 1 ng/mL doxycycline and were treated with 29 μM CCCP for 2 h. After treatment with DSP, the cells were incubated with RIPA buffer for protein extraction. Immunoprecipitation analysis was performed as described in the Methods. The experiments were repeated three times and representative data are shown. **c** *Left panel*, Immunoblot analysis of total proteins from siRNA-transfected HEK293 cells expressing Lon WT-3xFLAG. *Right panel*, Immunoprecipitation analysis was performed as described in the Methods. The experiments were repeated twice and representative data are shown. **d** TIMM22 protein was immunoprecipitated using the anti-TIMM22 antibody and protein G

magnetic beads. TIMM29 and AGK have been reported to associate with TIMM22. Immunoprecipitation analysis was performed as described above. The experiments were repeated twice and representative data are shown.

Supplementary Fig. S10 Depletion of the mitochondrial processing peptidase using another set of siRNAs. a Analysis of proteins from HeLa cells transfected with siRNAs. Cells were retransfected 3 d after the first siRNA transfection. Cells were used for experiments 3 d after the second transfection. Immunoblot analysis was performed as described in the legend of Fig. 1a. The experiments were repeated twice and representative data are shown. **b** Left, Reticulocyte lysate-synthesized MRPL30-3xFLAG or SOD2-3xFLAG was incubated with isolated mitochondria for 0, 20, 40 and 60 min. The reaction mixtures were analyzed by immunoblotting with FLAG antibody. Band intensities of the unprocessed and the mature forms are shown. Right, Reticulocyte lysate-synthesized MRPL30-3xFLAG or SOD2-3xFLAG was incubated with isolated mitochondria for 20 min and mitochondria were collected by centrifugation. The mitochondria were extracted

with RIPA buffer after treatment with the reversible crosslinker, DSP. Immunoprecipitation analysis using anti-LONP1 antibody was performed as described in the Methods. The experiments were repeated twice and representative data are shown. **c** *Left*, Reticulocyte lysate-synthesized MRPL30-3xFLAG or SOD2-3xFLAG was incubated with isolated mitochondria from siRNA-transfected HeLa cells for 60 min. *Right.* Reticulocyte lysate-synthesized MRPL30-3xFLAG or SOD2-3xFLAG was incubated with isolated mitochondria for 60 min. Mitochondria were collected by centrifugation. Soluble (S) and insoluble (I) proteins from the mitochondria were prepared and immunoblot analysis was performed. The experiments were repeated twice and representative data are shown.

Supplementary Fig. S11 Analysis of aggregated proteins in *LONP1*-knockdown HeLa cells using different preparation methods. Analysis of proteins from HeLa cells transfected with siRNA against *LONP1* (siLon#1). Cells were retransfected 3 d after the first siRNA transfection and used in experiments 3 d after the second transfection. **a** The cells were lysed with a buffer containing NP-40, Triton X-100 (TX100) or n-dodecyl-β-D-

maltoside (DDM). Before the centrifugation step, only NP-40 lysates were sonicated. After the centrifugation, the insoluble fractions were treated with an equal volume of urea buffer. Each lane was loaded with total (T), soluble (S) or insoluble (I) proteins obtained from 10 μg of total protein extract. The detailed preparation methods are described in the Methods.
b The cells were lysed with a buffer containing NP-40, Triton X-100 (TX100) or n-dodecyl-β-D-maltoside (DDM). Before the centrifugation step, all lysates were sonicated. Each lane was loaded with total (T), soluble (S) or insoluble (I) proteins obtained from 10 μg of total protein extract. The detailed preparation methods are described in the Methods.

Supplementary Fig. S12 The ratio of the intrinsically disordered regions of mitochondrial proteins having higher solubility (0.7>) or lower solubility (<0.3) in LONP1 knockdown cells. Protein solubility was determined from the results of the immunoblot analysis using soluble and insoluble cell fractions (Fig. 1h). The intrinsically disordered ratio of the proteins was calculated by SPOT-Disorder2. (Hanson et al., 2018)

Supplementary Fig. S13 Unprocessed blot/gel images corresponding to Figure 1.

Supplementary Fig. S14 Unprocessed blot/gel images corresponding to Figure 2.

Supplementary Fig. S15 Unprocessed blot/gel images corresponding to Figure 3.

Supplementary Fig. S16 Unprocessed blot/gel images corresponding to Figure 4.

Supplementary Fig. S17 Unprocessed blot images corresponding to Figure 5.

Supplementary Fig. S18 Unprocessed blot images corresponding to Figure 6.

Supplementary Fig. S19 Unprocessed blot images corresponding to Figure 7.

Supplementary Figure S1

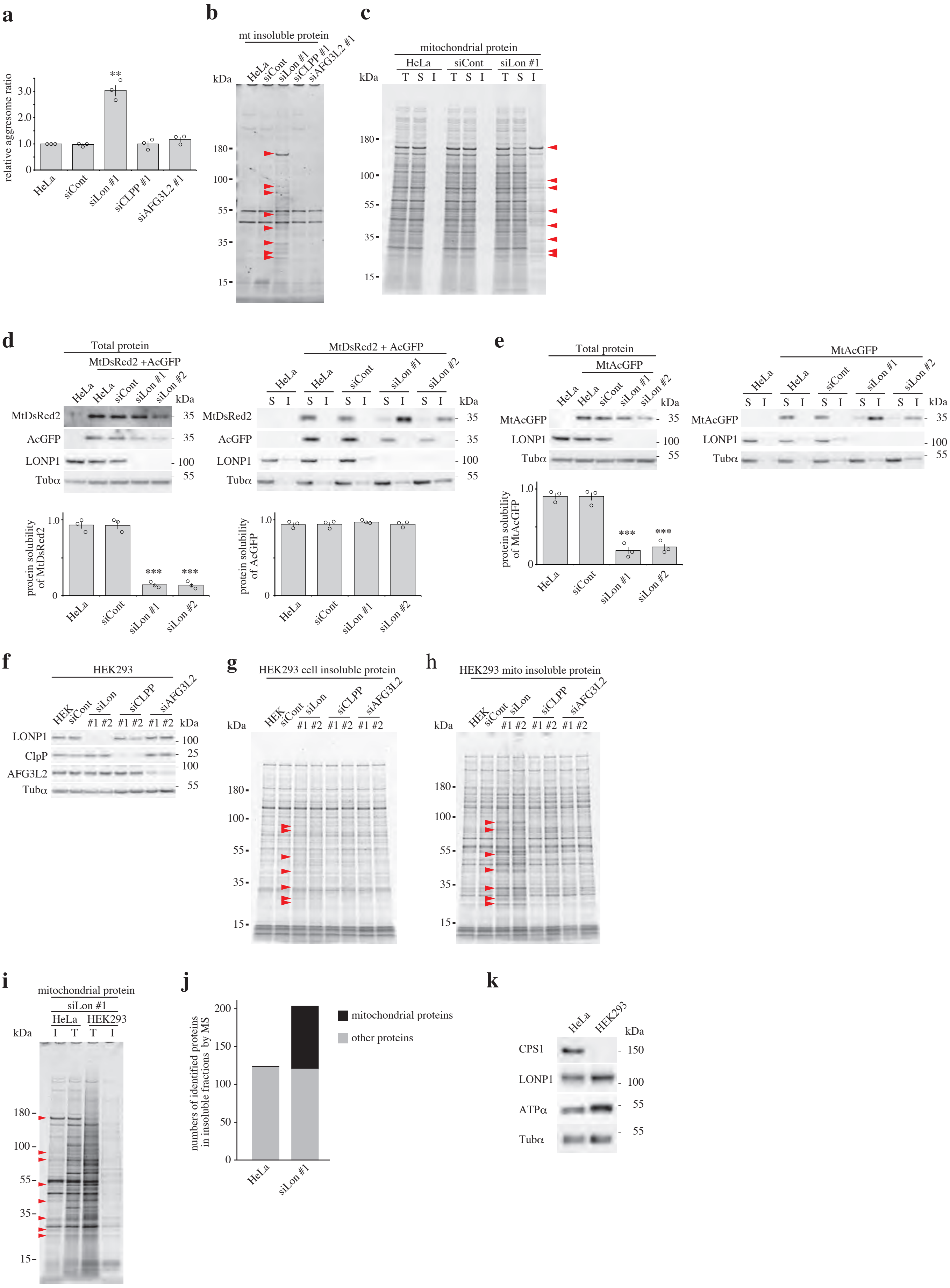

Supplementary Figure S2

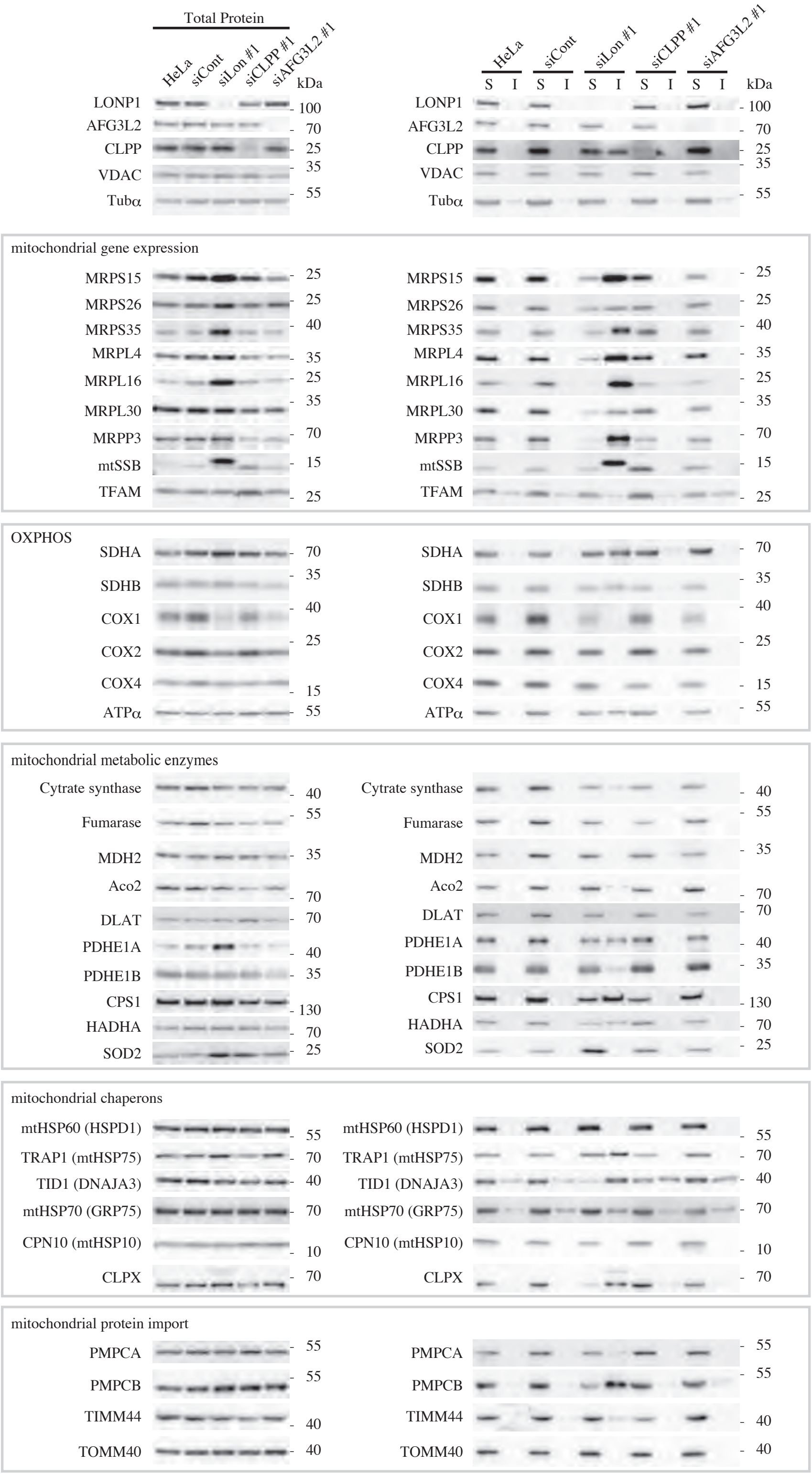

Supplementary Figure S3

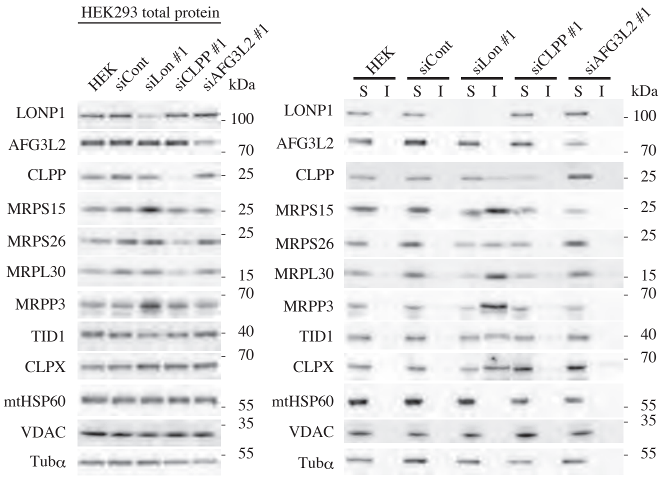

Supplementary Figure S4

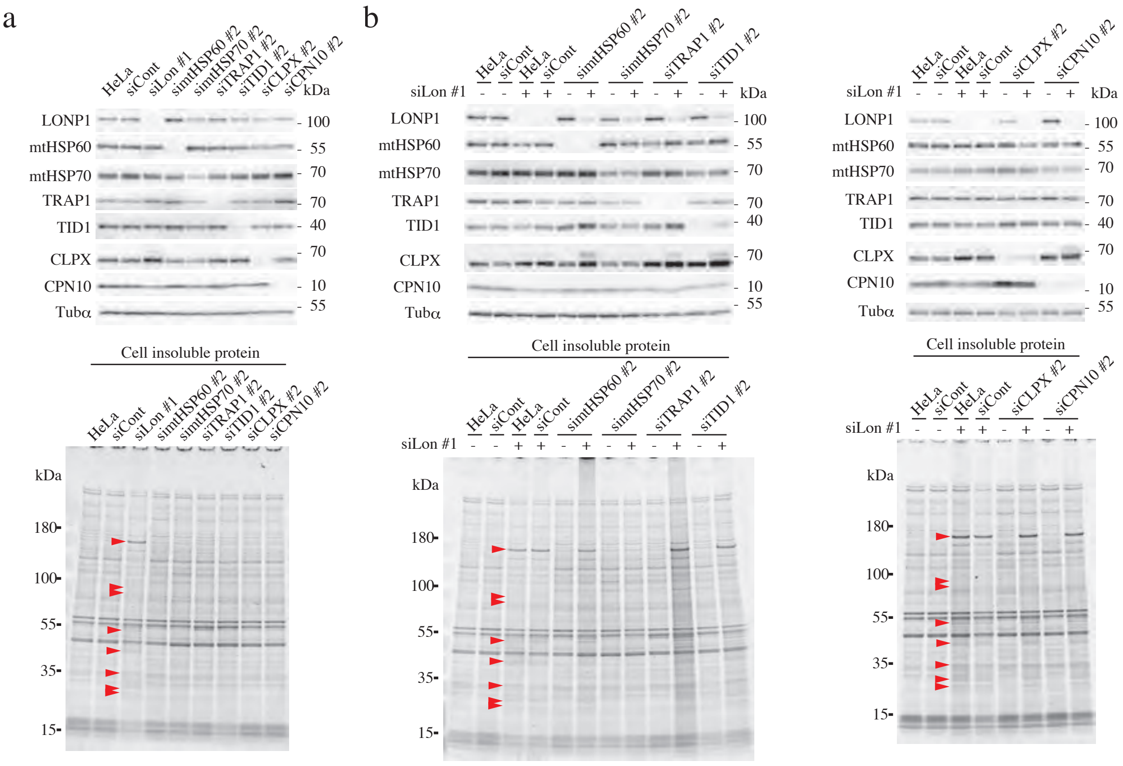

Supplementary Figure S5

a

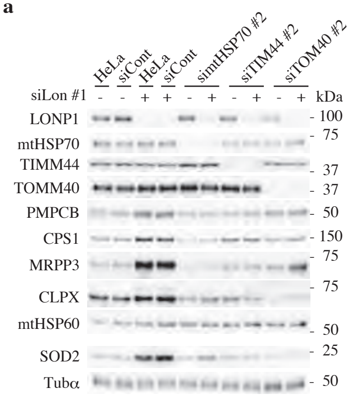

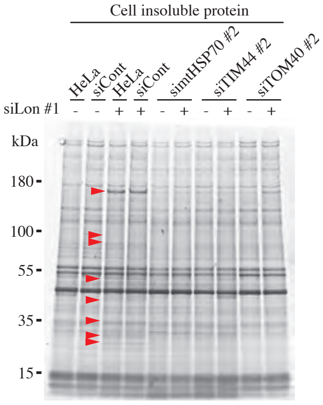

b

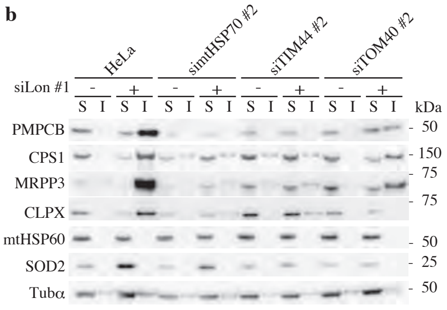

Supplementary Figure S6

a

```
   1 ATGGCCGCCTCTACAGGATACGTTAGACTGTGGGGAGCCGCCAGATGTTGGGTGCTGAGAAGGCCTATGCTTGCCGCTGCTGGTGGCAGA
  91 GTTCCTACAGCTGCTGGCGCTTGGCTGCTGAGAGGACAGAGAACCTGTGACGCCTCTCCTCCTTGGGCTCTGTGGGGAAGAGGACCTGCT
 181 ATTGGCGGACAGTGGCGCGGATTTTGGGAAGCCTCTTCTAGAGGCGGAGGCGCCTTTTCTGGCGGAGAGGATGCTTCTGAAGGCGGAGCT
 271 GAAGAAGGTGCTGGCGGAGCAGGCGGATCTGCAGGCGCTGGCGAAGGACCTGTTATCACAGCCCTGACACCTATGACAATCCCCGACGTG
 361 TTCCCTCATCTGCCCCTGATCGCCATCACCAGAAATCCCGTGTTTCCCCGGTTCATCAAGATCATCGAAGTGAAGAACAAGAAGCTGGTC
 451 GAGCTGCTGCGGCGGAAAGTTCGACTGGCTCAGCCTTATGTGGGCGTGTTCCTGAAGCGGGACGACAGCAATGAGAGCGACGTGGTGGAA
 541 AGCCTGGACGAGATCTACCACACCGGCACCTTCGCGCAAATTCACGAGATGCAGGACCTTGGGGACAAGCTGCGCATGATCGTCATGGGA
 631 CACAGAAGAGTCCATATCAGCAGACAGCTGGAGGTGGAGCCCGAGGAGCCGGAGGCGGAGAACAAGCACAAGCCCCGCAGGAAGTCAAAG
 721 CGGGGCAAGAAGGAGGCGGAGGACGAGCTGAGCGCCAGGCACCCGGCGGAGCTGGCGATGGAGCCCACCCCTGAGCTCCCGGCTGAGGTG
 811 CTCATGGTGGAGGTAGAGAACGTTGTCCACGAGGACTTCCAGGTCACGGAGGAGGTGAAAGCCCTGACTGCAGAGATCGTGAAGACCATC
 901 CGGGACATCATTGCCTTGAACCCTCTCTACAGGGAGTCAGTGCTGCAGATGATGCAGGCTGGCCAGCGGGTGGTGGACAACCCCATCTAC
 991 CTGAGCGACATGGGCGCCGCGCTCACCGGGGCCGAGTCCCATGAGCTGCAGGACGTCCTGGAAGAGACTAACATCCCGAAACGCCTGTAC
1081 AAGGCCCTCTCCCTGCTGAAGAAGGAATTTGAACTGAGCAAGCTGCAGCAGCGCCTGGGGCGGGAGGTGGAGGAGAAGATCAAGCAGACC
1171 CACCGTAAGTACCTGCTGCAGGAGCAGCTAAAGATCATCAAGAAGGAGCTGGGCCTGGAGAAGGACGACAAGGATGCCATCGAGGAGAAG
1261 TTCCGGGAGCGCCTGAAGGAGCTCGTGGTCCCCAAGCACGTCATGGATGTTGTGGACGAGGAGCTGAGCAAGCTGGGCCTGCTGGACAAC
1351 CACTCCTCGGAGTTCAATGTCACCCGCAACTACCTAGACTGGCTCACGTCCATCCCTTGGGGCAAGTACAGCAACGAGAACCTGGACCTG
1441 GCGCGGGCACAGGCAGTGCTGGAGGAAGACCACTACGGCATGGAGGACGTCAAGAAACGCATCCTGGAGTTCATTGCCGTTAGCCAGCTC
1531 CGCGGCTCCACCCAGGGCAAGATCCTCTGCTTCTATGGCCCCCCTGGCGTGGGTAAGACCAGCATTGCTCGCTCCATCGCCCGCGCCCTG
1621 AACCGAGAGTACTTCCGCTTCAGCGTCGGGGGCATGACTGACGTGGCTGAGATCAAGGGCCACAGGCGGACCTACGTGGGCGCCATGCCC
1711 GGGAAGATCATCCAGTGTTTGAAGAAGACCAAGACGGAGAACCCCCTGATCCTCATCGACGAGGTGGACAAGATCGGCCGAGGCTACCAG
1801 GGGGACCCGTCGTCGGCACTGCTGGAGCTGCTGGACCCAGAGCAGAATGCCAACTTCCTGGACCACTACCTGGACGTGCCCGTGGACTTG
1891 TCCAAGGTGCTGTTCATCTGCACGGCCAACGTCACGGACACCATCCCCGAGCCGCTGCGAGACCGTATGGAGATGATCAACGTGTCGGGC
1981 TACGTGGCCCAGGAGAAGCTGGCCATTGCGGAGCGCTACCTGGTGCCCCAGGCTCGCGCCCTGTGTGGCTTGGATGAGAGCAAGGCCAAG
2071 CTGTCATCGGACGTGCTGACGCTGCTCATCAAGCAGTACTGCCGCGAGAGCGGTGTCCGCAACCTGCAGAAGCAAGTGGAGAAGGTGTTA
2161 CGGAAATCGGCCTACAAGATTGTCAGCGGCGAGGCCGAGTCCGTGGAGGTGACGCCCGAGAACCTGCAGGACTTCGTGGGGAAGCCCGTG
2251 TTCACCGTGGAGCGCATGTATGACGTGACACCGCCCGGCGTGGTCATGGGGCTGGCCTGGACCGCAATGGGAGGCTCCACGCTGTTTGTG
2341 GAGACATCCCTGAGACGGCCACAGGACAAGGATGCCAAGGGTGACAAGGATGGCAGCCTGGAGGTGACAGGCCAGCTGGGGGAGGTGATG
2431 AAGGAGAGCGCCCGCATAGCCTACACCTTCGCCAGAGCCTTCCTCATGCAGCACGCCCCCGCCAATGACTACCTGGTGACCTCACACATC
2521 CACCTGCATGTGCCCGAGGGCGCCACCCCCAAGGACGGCCCAAGCGCAGGCTGCACCATCGTCACGGCCCTGCTGTCCCTGGCCATGGGC
2611 AGGCCTGTCCGGCAGAATCTGGCCATGACTGGCGAAGTCTCCCTCACGGGCAAGATCCTGCCTGTTGGTGGCATCAAGGAGAAGACCATT
2701 GCGGCCAAGCGCGCAGGGGTGACGTGCATCGTCCTGCCAGCGGAAAATAAGAAAGATTTCTACGACCTGGCAGCCTTCATCACCGAGGGC
2791 CTGGAGGTGCACTTCGTGGAACACTACCGGGAGATCTTCGACATCGCCTTCCCGGACGAGCAGGCAGAGGCGCTGGCCGTGGAACGGGAT
2881 TACAAGGATCACGATGGCGACTATAAGGATCACGACATCGACTACAAGGACGATGACGACAAATGA
```

b

LONP1 siRNA #1 target sequence

```
LONP1 cDNA      ccaatattcctaagcggct
                 *  *  *  *  *  *
codon-changed  CTAACATCCCGAAACGCCT
```

c

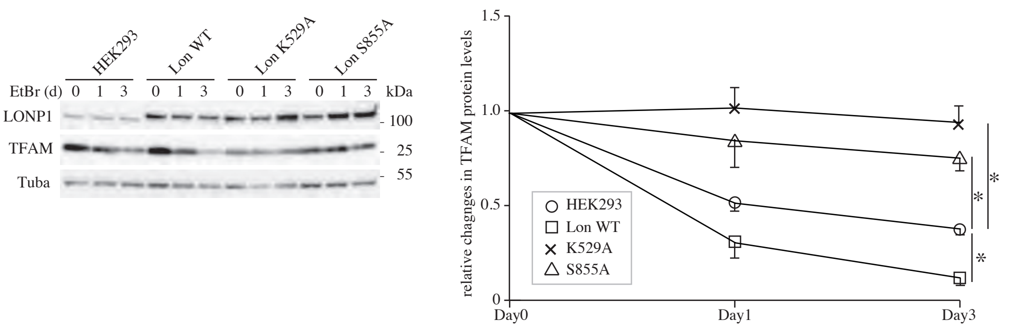

Supplementary Figure S7

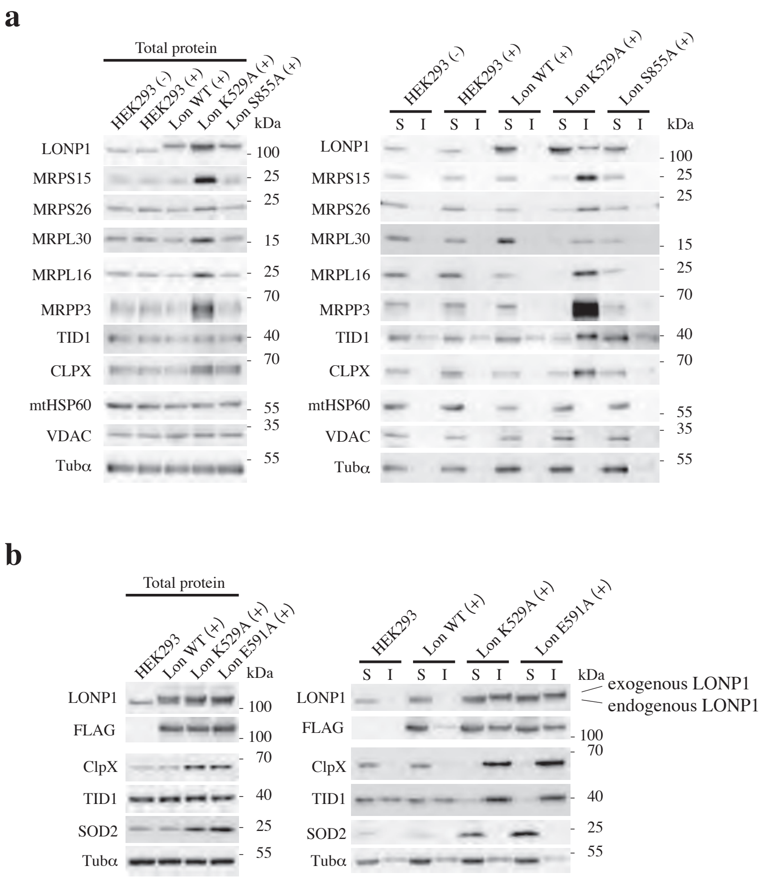

Supplementary Figure S8

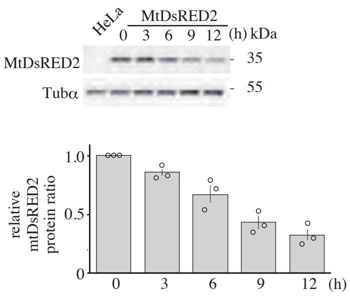

Supplementary Figure S9

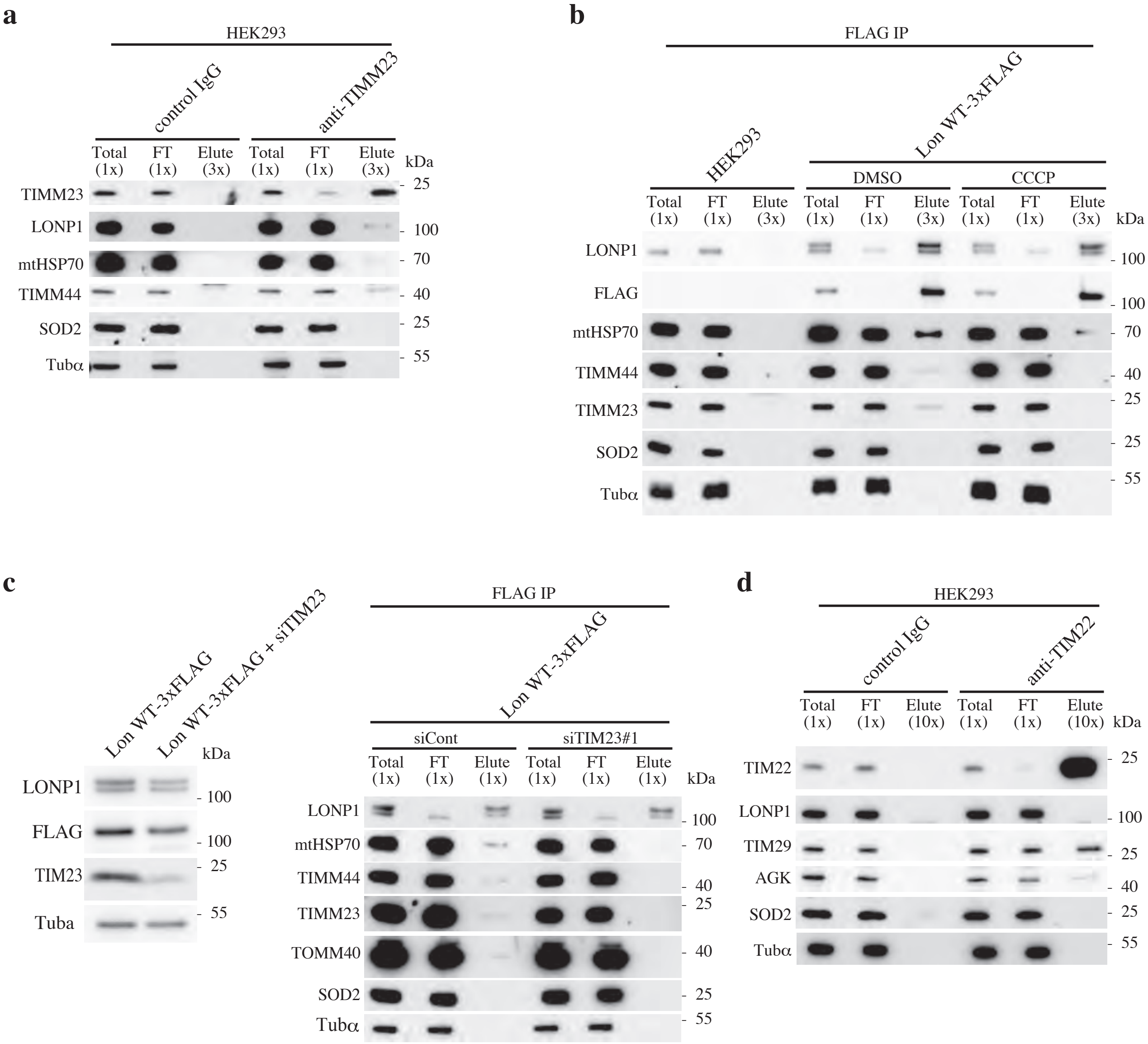

Supplementary Figure S10

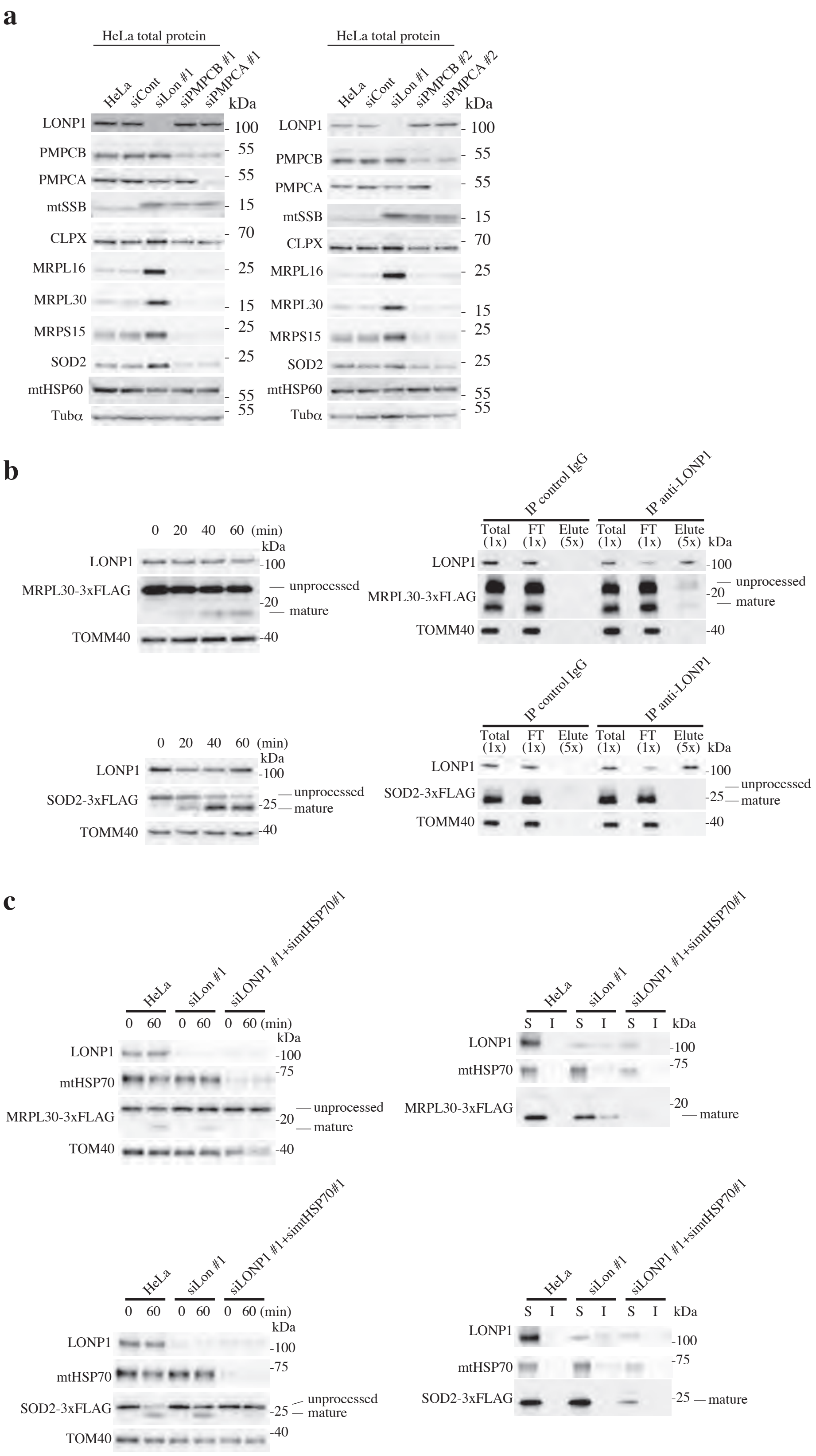

Supplementary Figure S11

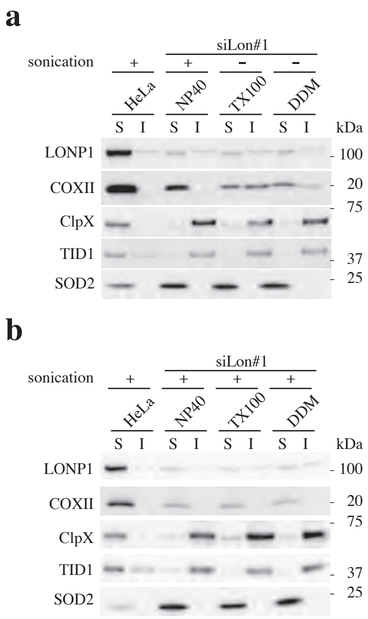

Supplementary Figure S12

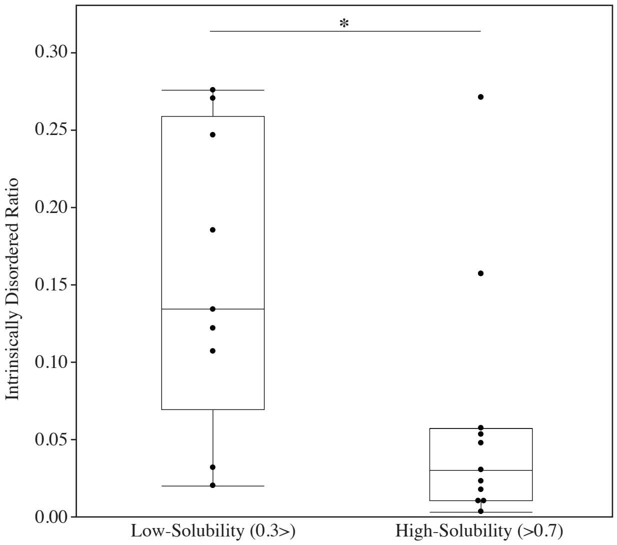

Supplementary Figure S13

a Blot images corresponding to Figure 1a

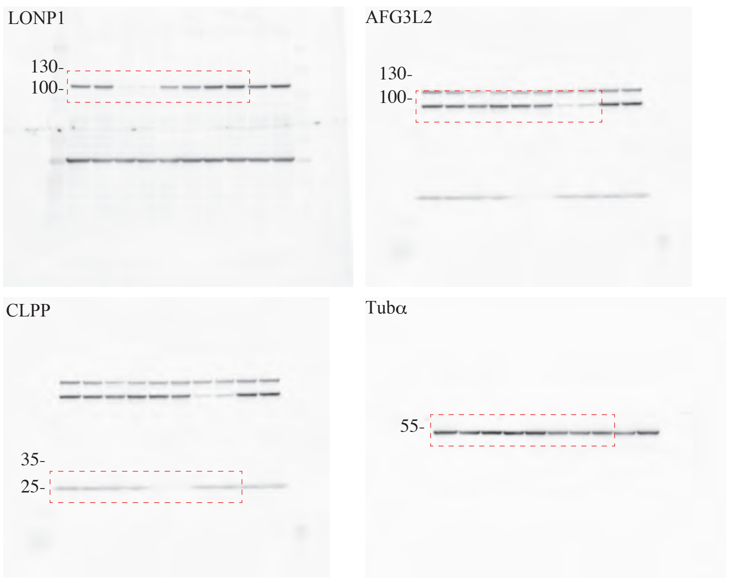

b Gel image corresponding to Figure 1d

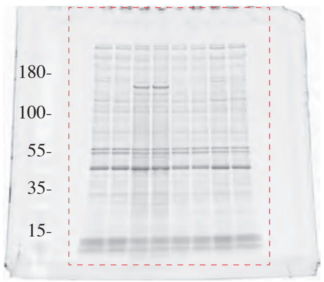

c Gel image corresponding to Figure 1e

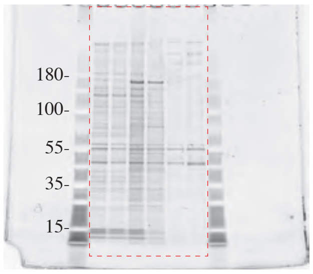

d Gel image corresponding to Figure 1f

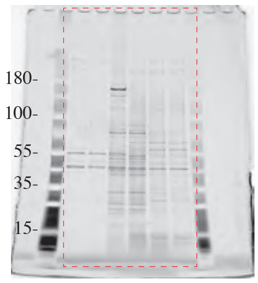

Supplementary Figure S14

a Blot/Gel images corresponding to Figure 2a

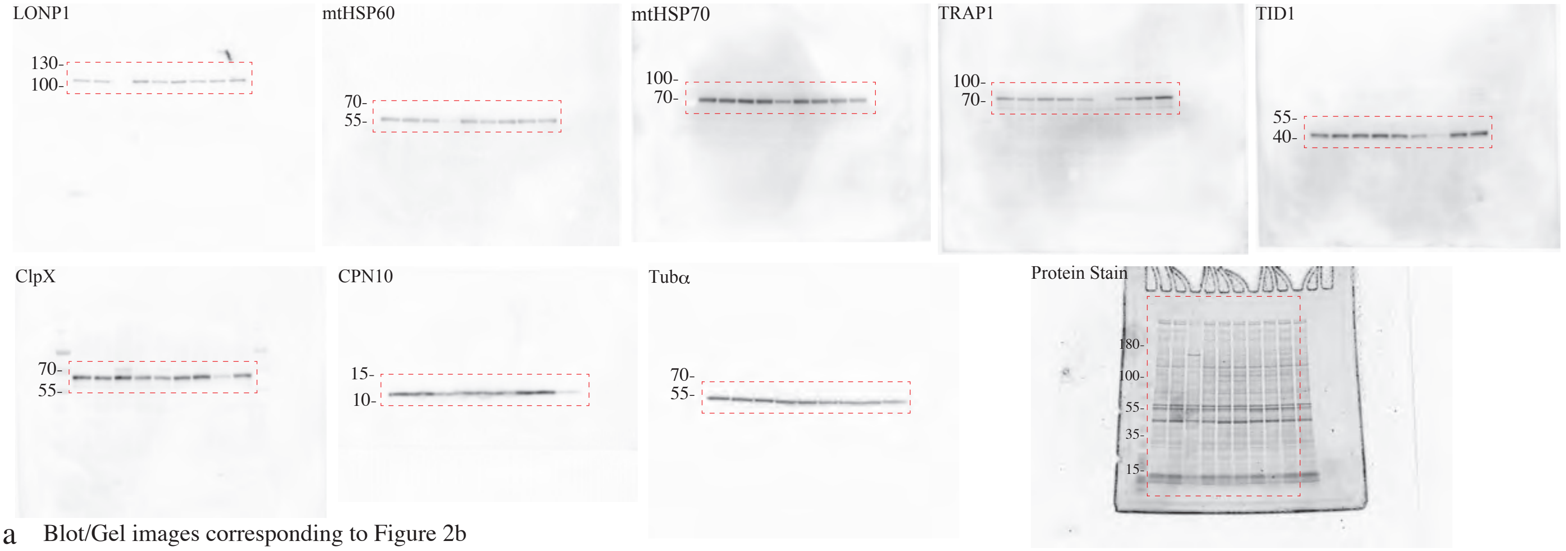

a Blot/Gel images corresponding to Figure 2b

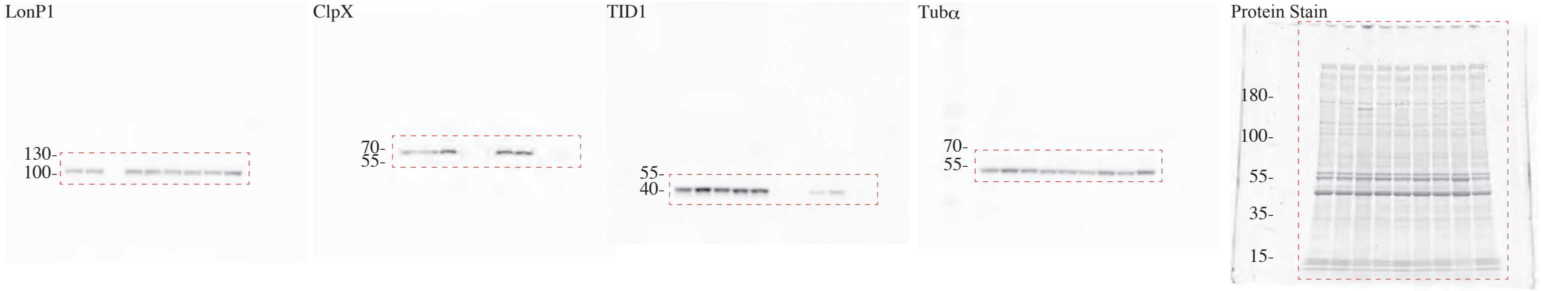

a Blot/Gel images corresponding to Figure 2c

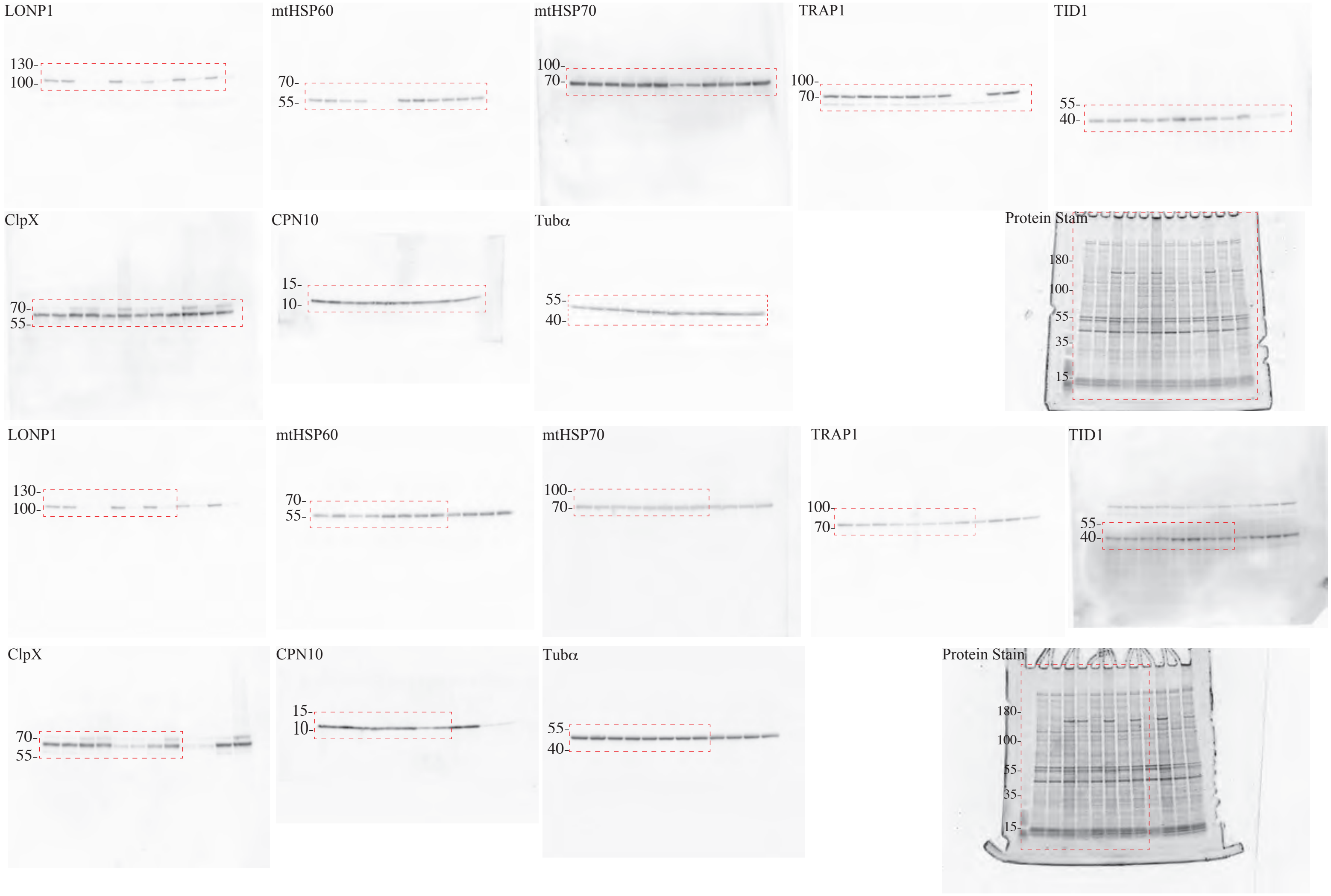

Supplementary Figure S15

a Blot/Gel images corresponding to Figure 3b

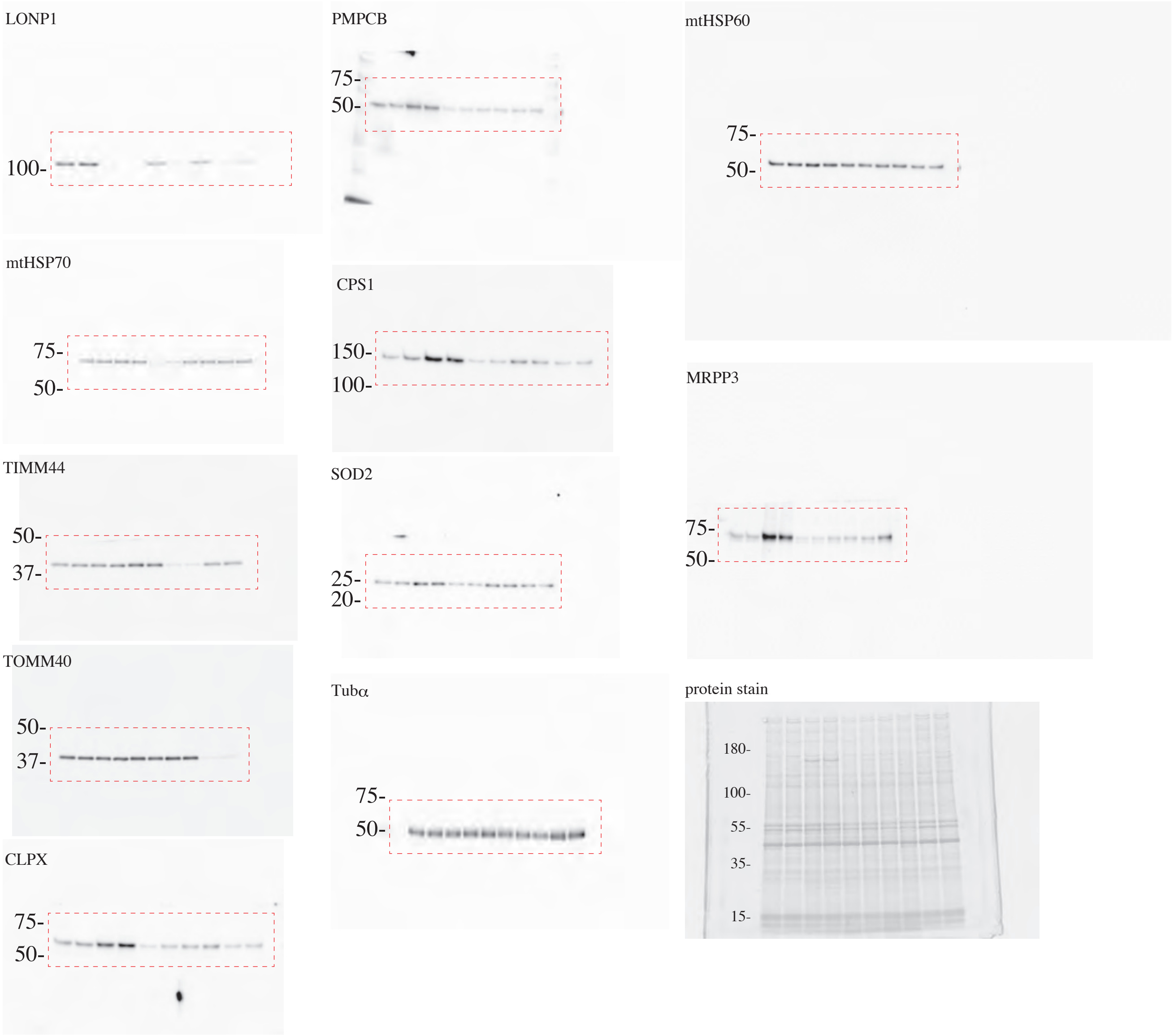

b Blot images corresponding to Figure 3c

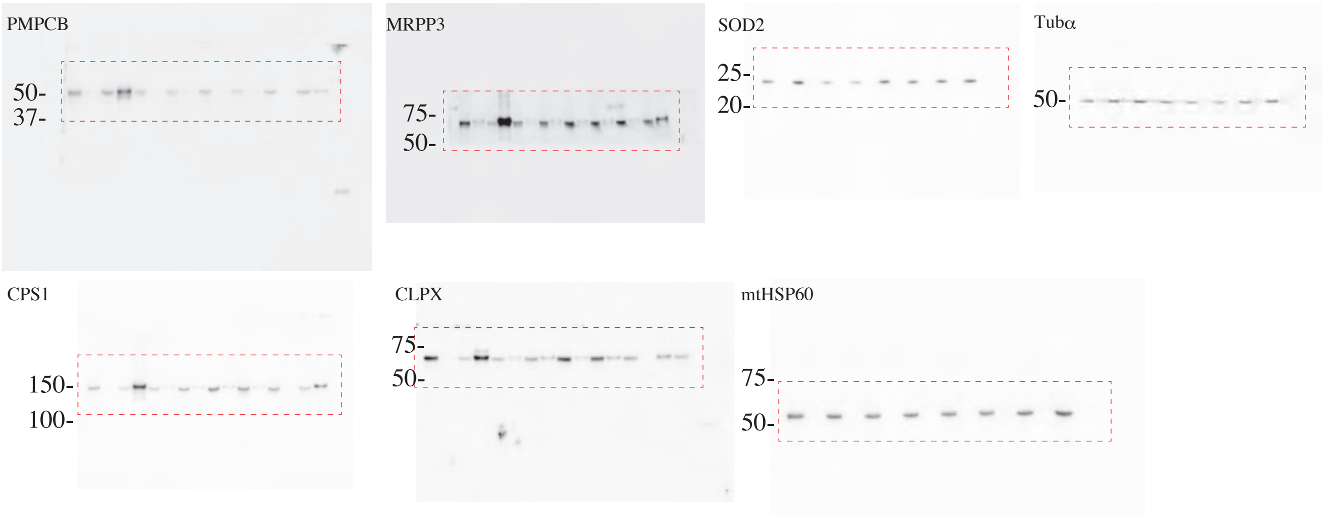

Supplementary Figure S16

a Blot/Gel images corresponding to Figure 4b

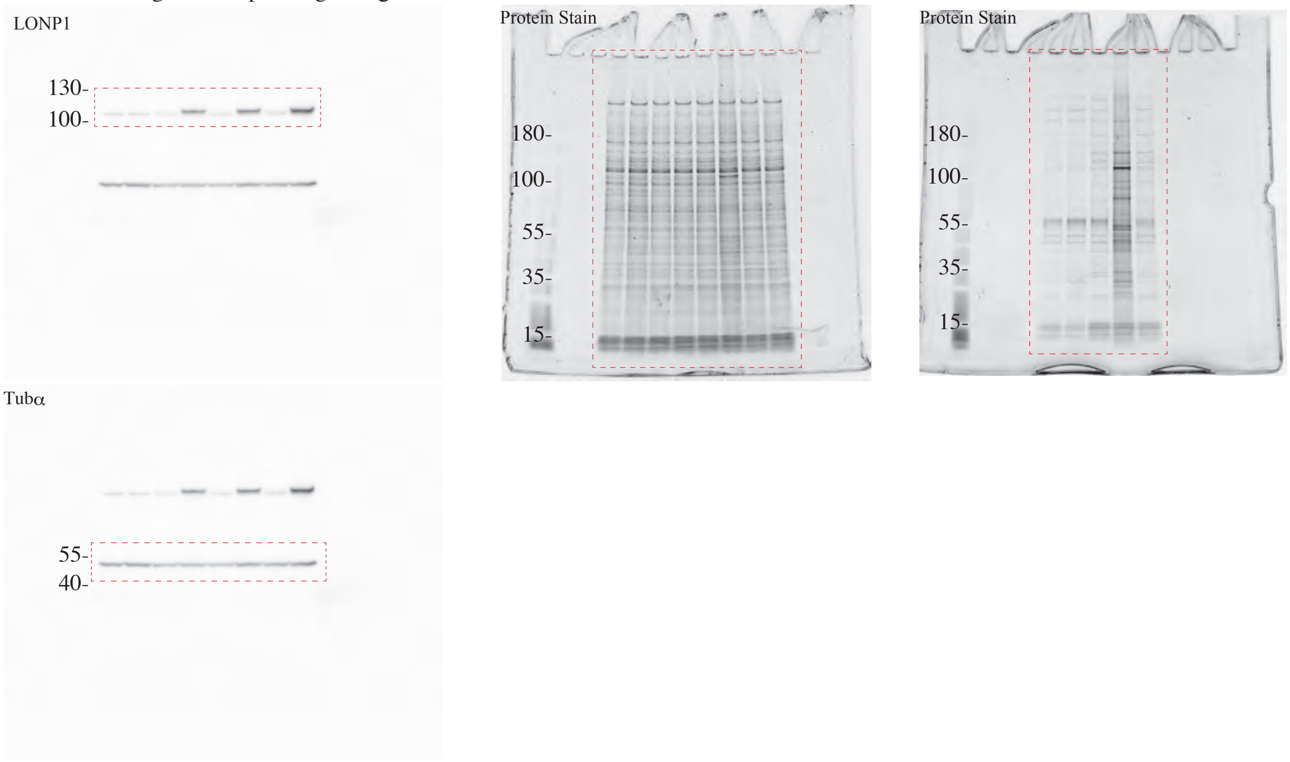

b Blot/Gel images corresponding to Figure 4c

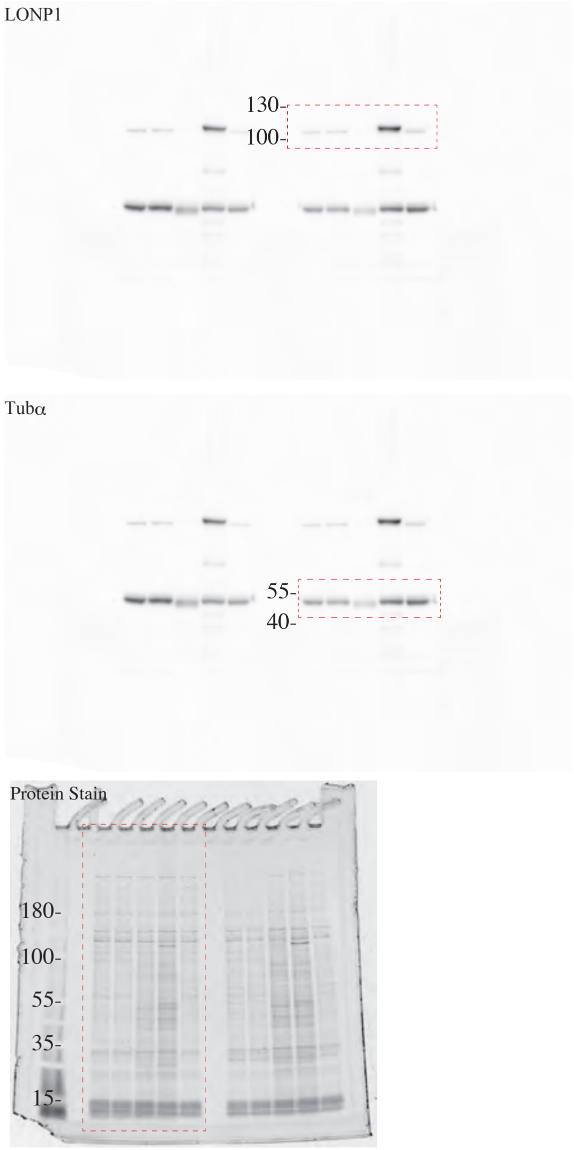

c Blot/Gel images corresponding to Figure 4d

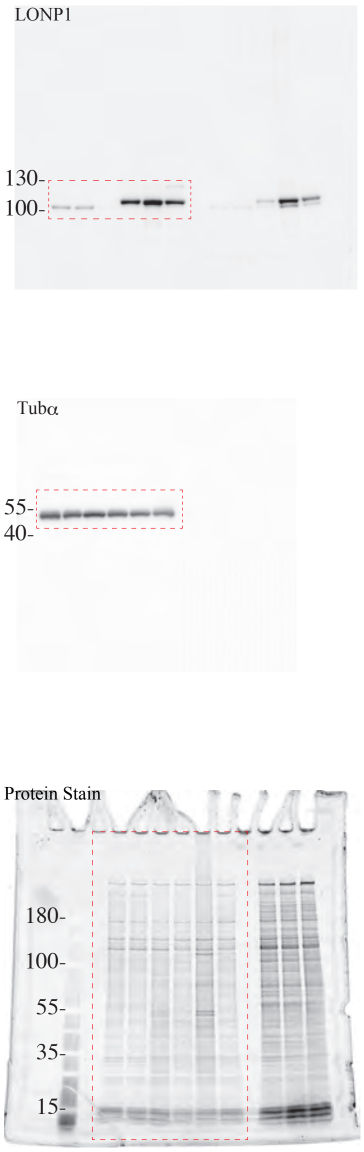

Supplementary Figure S17

a Blot images corresponding to Figure 5a

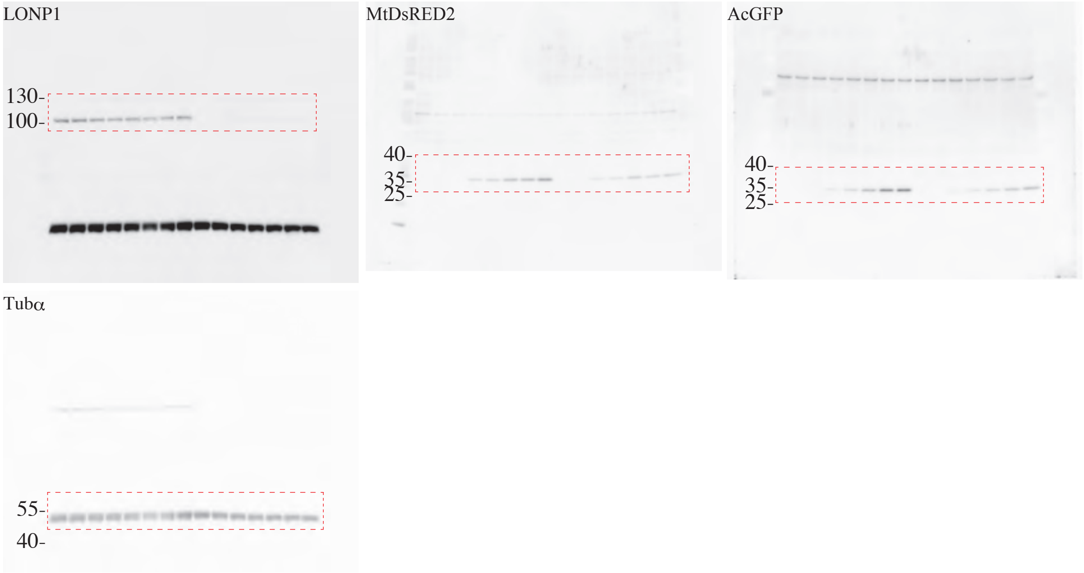

b Blot images corresponding to Figure 5b

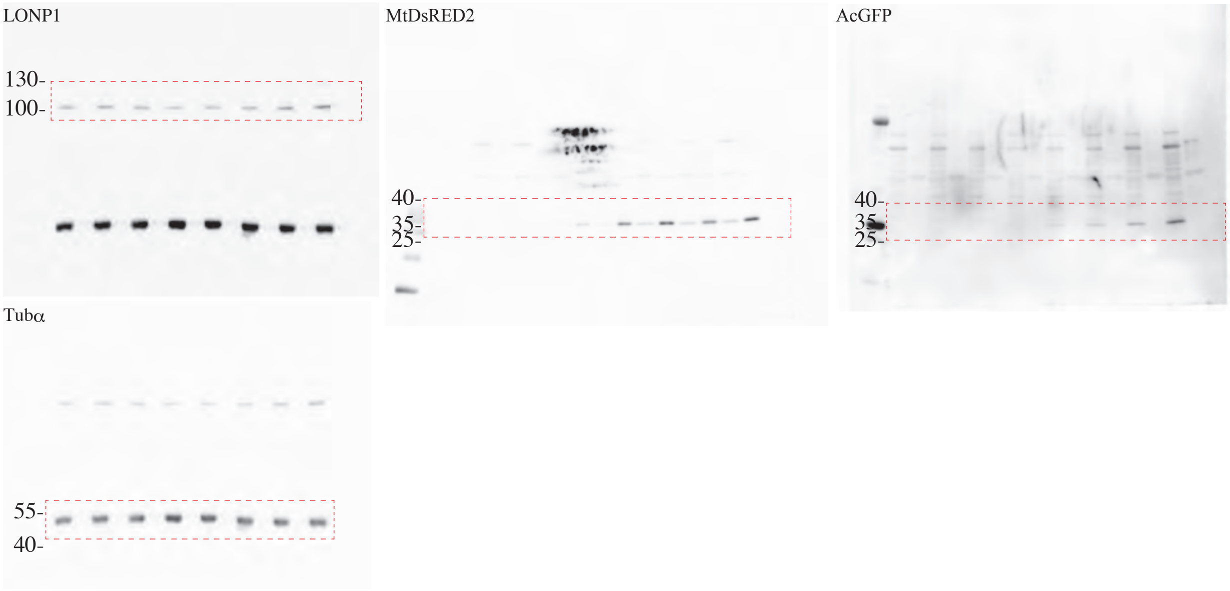

c Blot images corresponding to Figure 5c

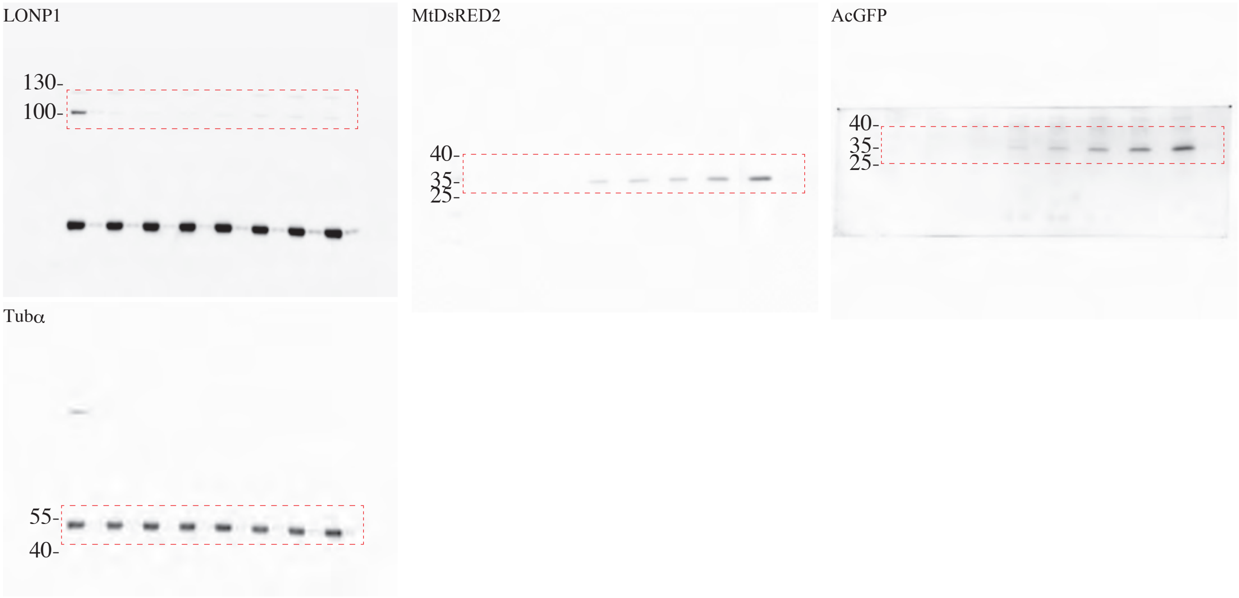

Supplementary Figure S18

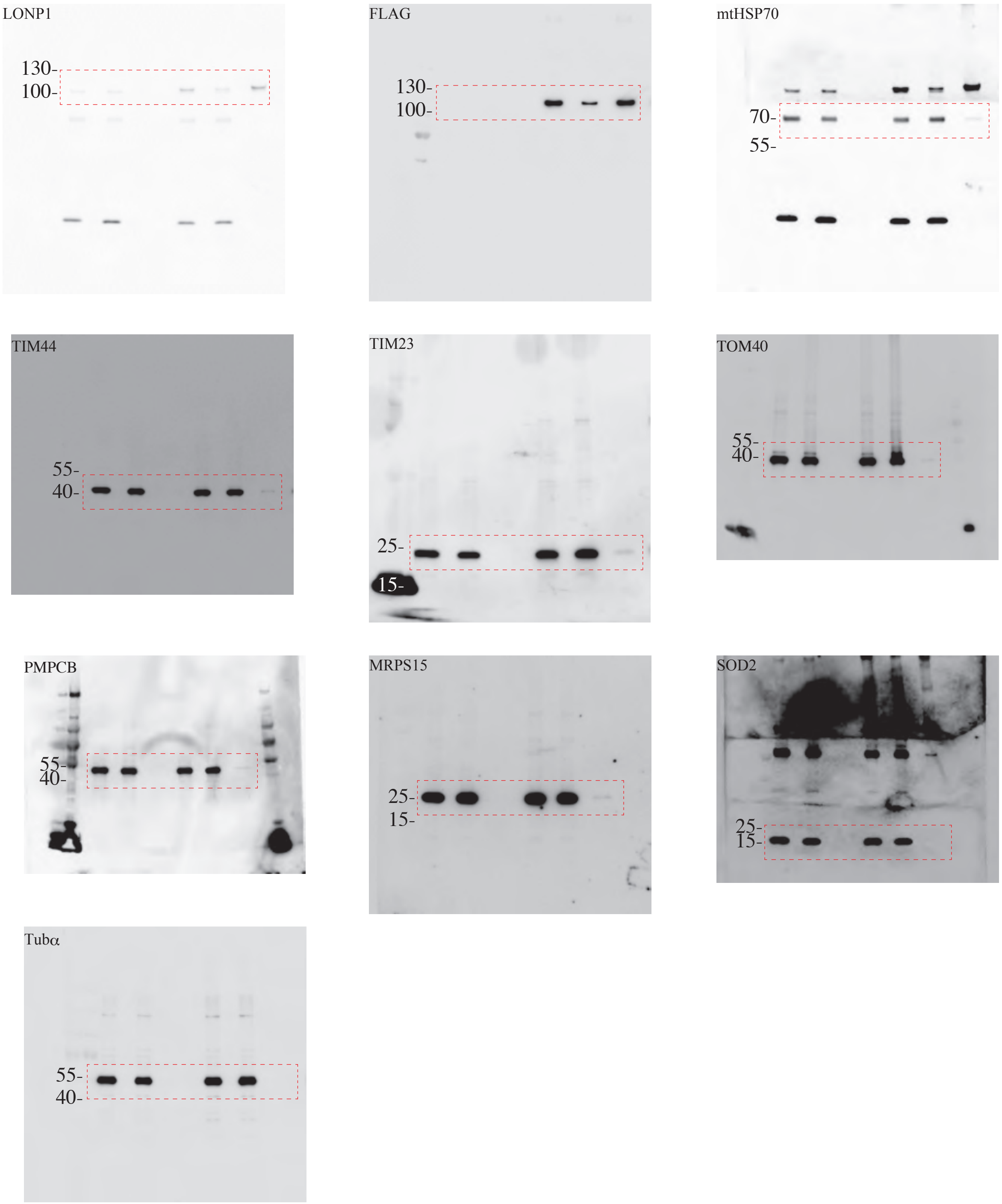

Supplementary Figure S19

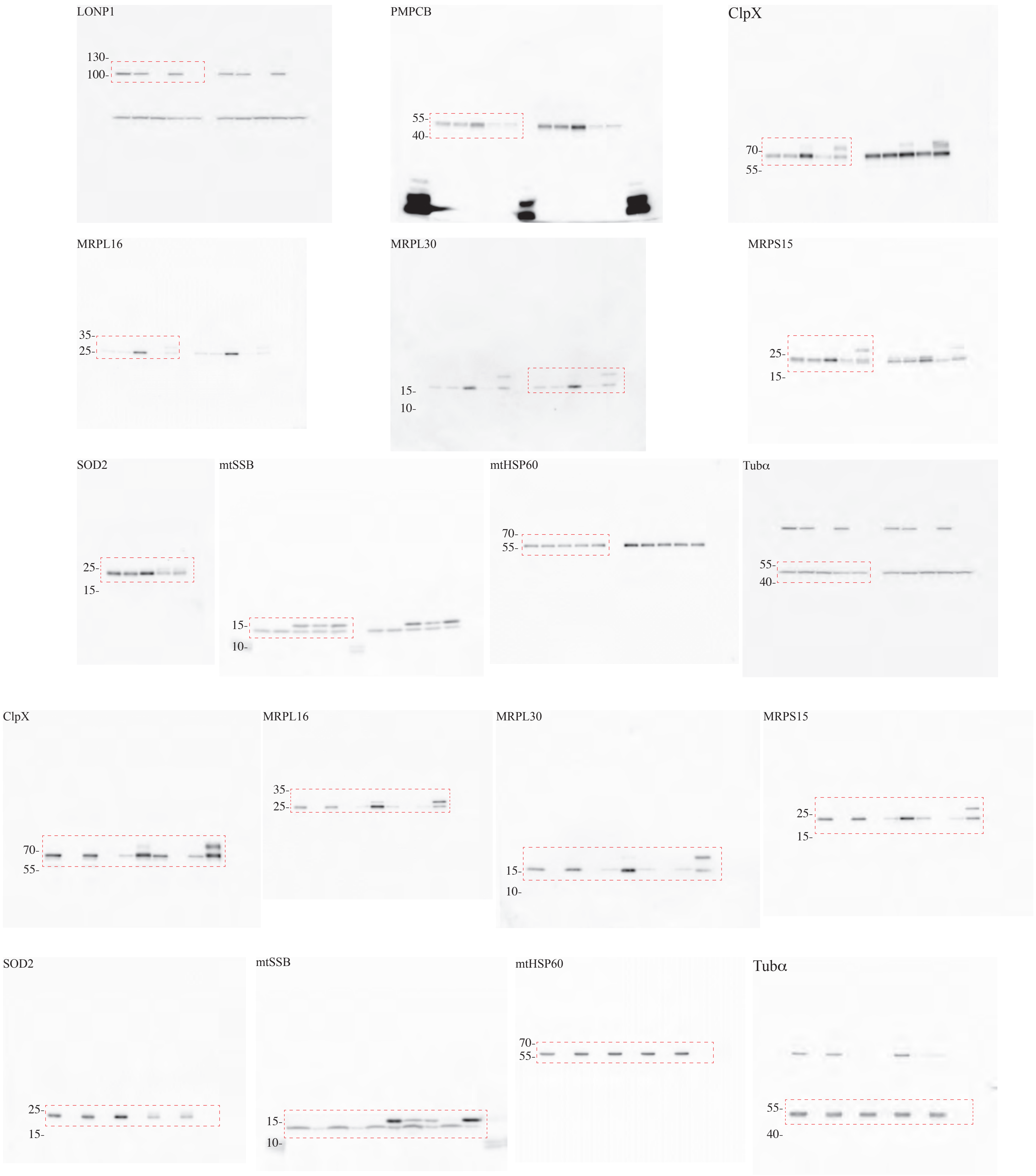

Supplement: Supplementary file 1 — Supplementary Information [file 42003_2021_2498_MOESM1_ESM.pdf]
