# Supplementary material for: Mitochondrial Lon protease is a gatekeeper for proteins newly imported into the matrix
Source: Commun Biol. 2021 Aug 16;4:974. doi: 10.1038/s42003-021-02498-z (PMC8368198; doi:10.1038/s42003-021-02498-z)
Supplement: Supplementary file 2 — Description of Additional Supplementary Files [file 42003_2021_2498_MOESM2_ESM.pdf]

File Name: Supplementary Data 1.

Description: Mass spectrometry analysis of the aggregated mitochondrial proteins from LONP1 knockdown HeLa cells. The list of the aggregated mitochondrial proteins from LONP1 knockdown HeLa cells is available as a Supplementary Data 1.

File Name: Supplementary Data 2.

Description: Mass spectrometry analysis of the aggregated mitochondrial proteins from LONP1 knockdown HEK293 cells. The list of the aggregated mitochondrial proteins from LONP1 knockdown HEK293 cells is available as a Supplementary Data 2.

File Name: Supplementary Data 3.

Description: The list of materials used in experiments is available as a Supplementary Data 3.

File Name: Supplementary Data 4.

Description: Source data for the graphs is available as a Supplementary Data 4.
