# Supplementary material for: Mitochondrial Lon protease is a gatekeeper for proteins newly imported into the matrix
Source: Commun Biol. 2021 Aug 16;4:974. doi: 10.1038/s42003-021-02498-z (PMC8368198; doi:10.1038/s42003-021-02498-z)
Supplement: Supplementary file 7 — Reporting Summary [file 42003_2021_2498_MOESM7_ESM.pdf]

nature research

Corresponding author(s): Yuichi Matsushima

Last updated by author(s): Jul 19, 2021

Reporting Summary

Nature Research wishes to improve the reproducibility of the work that we publish. This form provides structure for consistency and transparency in reporting. For further information on Nature Research policies, see our Editorial Policies and the Editorial Policy Checklist.

Statistics

For all statistical analyses, confirm that the following items are present in the figure legend, table legend, main text, or Methods section.

n/a	Confirmed	
☐	☒	The exact sample size (n) for each experimental group/condition, given as a discrete number and unit of measurement
☐	☒	A statement on whether measurements were taken from distinct samples or whether the same sample was measured repeatedly
☐	☒	The statistical test(s) used AND whether they are one- or two-sided<br>*Only common tests should be described solely by name; describe more complex techniques in the Methods section.*
☒	☐	A description of all covariates tested
☒	☐	A description of any assumptions or corrections, such as tests of normality and adjustment for multiple comparisons
☐	☒	A full description of the statistical parameters including central tendency (e.g. means) or other basic estimates (e.g. regression coefficient) AND variation (e.g. standard deviation) or associated estimates of uncertainty (e.g. confidence intervals)
☐	☒	For null hypothesis testing, the test statistic (e.g. F, t, r) with confidence intervals, effect sizes, degrees of freedom and P value noted<br>*Give P values as exact values whenever suitable.*
☒	☐	For Bayesian analysis, information on the choice of priors and Markov chain Monte Carlo settings
☒	☐	For hierarchical and complex designs, identification of the appropriate level for tests and full reporting of outcomes
☒	☐	Estimates of effect sizes (e.g. Cohen's d, Pearson's r), indicating how they were calculated

Our web collection on statistics for biologists contains articles on many of the points above.

Software and code

Policy information about availability of computer code

Data collection	N/A
Data analysis	N/A

For manuscripts utilizing custom algorithms or software that are central to the research but not yet described in published literature, software must be made available to editors and reviewers. We strongly encourage code deposition in a community repository (e.g. GitHub). See the Nature Research guidelines for submitting code & software for further information.

Data

Policy information about availability of data

All manuscripts must include a data availability statement. This statement should provide the following information, where applicable:

- Accession codes, unique identifiers, or web links for publicly available datasets
- A list of figures that have associated raw data
- A description of any restrictions on data availability

All the data used in this study are available from the corresponding author upon reasonable request.

Field-specific reporting

Please select the one below that is the best fit for your research. If you are not sure, read the appropriate sections before making your selection.

☒ Life sciences ☐ Behavioural & social sciences ☐ Ecological, evolutionary & environmental sciences

For a reference copy of the document with all sections, see nature.com/documents/nr-reporting-summary-flat.pdf

Life sciences study design

All studies must disclose on these points even when the disclosure is negative.

Sample size	Sample size was determined based on similar studies in this field.
Data exclusions	No data exclusions carried out.
Replication	The reproducibility of all experimental findings in this study was confirmed.
Randomization	Randomization of the data was not required.
Blinding	Blinding was not required.

Reporting for specific materials, systems and methods

We require information from authors about some types of materials, experimental systems and methods used in many studies. Here, indicate whether each material, system or method listed is relevant to your study. If you are not sure if a list item applies to your research, read the appropriate section before selecting a response.

Materials & experimental systems

n/a	Involved in the study	
☐	☒	Antibodies
☐	☒	Eukaryotic cell lines
☒	☐	Palaeontology and archaeology
☒	☐	Animals and other organisms
☒	☐	Human research participants
☒	☐	Clinical data
☒	☐	Dual use research of concern

Methods

n/a	Involved in the study	
☒	☐	ChIP-seq
☒	☐	Flow cytometry
☒	☐	MRI-based neuroimaging

Antibodies

Antibodies used

Detailed information about the antibodies used in this study are provided in Supplementary Data 3.

Validation

Anti-LONP1
Methods section in this manuscript
Anti-CLPP abcam CAT# ab124822
https://www.abcam.com/clpp-antibody-epr7133-ab124822.html
Anti-AFG3L2 Proteintech CAT# 14631-1-AP
https://www.ptglab.com/Products/AFG3L2-Antibody-14631-1-AP.htm
Anti-mtHSP70 CST CAT# 35935
https://www.cellsignal.com/products/primary-antibodies/grp75-d13h4-xp-rabbit-mab/3593
Anti-mtHSP60 SANTA CRUZ CAT# sc-59567
https://www.scbt.com/p/hsp-60-antibody-lk1?productCanUrl=hsp-60-antibody-lk1&_requestid=2382054
Anti-TID1 abcam CAT# ab181024
https://www.abcam.com/tid1-antibody-epr12414-ab181024.html?#description_protocols
Anti-TRAP1 abcam CAT# ab109323
https://www.abcam.com/trap1-antibody-epr5381-ab109323.html
Anti-CPN10 abcam CAT# ab108600
https://www.abcam.com/epf-antibody-epr4476-ab108600.html
Anti-CLPX abcam CAT# ab168338
https://www.abcam.com/clpx-antibody-ep8772-ab168338.html
Anti-PMPCA SANTA CRUZ CAT# sc-390718
https://www.scbt.com/p/pmpca-antibody-f-4?productCanUrl=pmpca-antibody-f-4&_requestid=2382531
Anti-PMPCB Proteintech CAT# 16064-1-AP
https://www.ptglab.com/Products/PMPCB-Antibody-16064-1-AP.htm
Anti-TOMM40 Proteintech CAT# 18409-1-AP

https://www.ptglab.com/Products/TOMM40-Antibody-18409-1-AP.htm
Anti-TIMM22 Proteintech CAT# 14927-1-AP
https://www.ptglab.co.jp/products/TIMM22-Antibody-14927-1-AP.htm
Anti-TIMM29 (C19orf52) Proteintech CAT# 25652-1-AP
https://www.ptglab.co.jp/products/C19orf52-Antibody-25652-1-AP.htm
Anti-AGK GeneTex CAT# GTX107413
https://www.genetex.com/Product/Detail/Acylglycerol-kinase-antibody/GTX107413
Anti-TIMM44 abcam CAT# ab194829
https://www.abcam.com/tim44-antibody-epr16821-ab194829.html
Anti-TIMM23 Proteintech CAT# 11123-1-AP
https://www.ptglab.com/Products/TIMM23-Antibody-11123-1-AP.htm
Living Colors Av. Peptide Antibody (AcGFP) TAKARA CAT# 632377
https://www.takarabio.com/assets/documents/Certificate%20of%20Analysis/632377-PA923065.pdf
Anti-DsRED TAKARA CAT# 632496
https://www.takarabio.com/products/antibodies-and-elisa/fluorescent-protein-antibodies/red-fluorescent-protein-antibodies?catalog=632496
anti-DDDDK (FLAG) MBL CAT# PM020
https://ruo.mbl.co.jp/bio/e/dtl/A/?pcd=PM020
Anti-VDAC Proteintech CAT# 63345-1-Ig
https://www.ptglab.com/products/VDAC1-Porin-Antibody-66345-1-Ig.htm
Anti-Tuba MBL CAT# PM054
https://ruo.mbl.co.jp/bio/e/dtl/A/?pcd=PM054
Anti-MRPS15 abcam CAT# ab137070
https://www.abcam.com/mrps15-antibody-epr9361-ab137070.html
Anti-MRPS26 abcam CAT# ab181863
https://www.abcam.com/mrps26-antibody-epr14396-ab181863.html
Anti-MRPS35 abcam CAT# ab182160
https://www.abcam.com/mrps35-antibody-epr117312-ab182160.html
Anti-MRPL4 abcam CAT# ab180165
https://www.abcam.com/mrpl4-antibody-epr13151-ab180165.html
Anti-MRPL16 abcam CAT# ab181834
https://www.abcam.com/mrpl16-antibody-epr14351-ab181834.html
Anti-MRPL30 abcam CAT# ab179819
https://www.abcam.com/mrpl30-antibody-epr12502-ab179819.html
Anti-POLRMT SANTA CRUZ CAT# sc-365082
https://www.scbt.com/p/mtrpol-antibody-b-1?requestFrom=search
Anti-TFB2M ABGENT CAT# AP10145b
http://www.abcepta.com/products/AP10145b-TFB2M-Antibody-C-term
Anti-MRPPP3 abcam CAT# ab185942
https://www.abcam.com/mrpp3-antibody-epr14321-30-ab185942.html
Anti-POLGA CST CAT# 13609S
https://www.cellsignal.jp/products/primary-antibodies/dna-polymerase-g-d1y6r-rabbit-mab/13609
Anti-mtSSB home made
Takamatsu, C., Umeda, S., Ohsato, T., Ohno, T., Abe, Y., Fukuoh, A., Shinagawa, H., Hamasaki, N., and Kang, D. (2002). Regulation of mitochondrial D-loops by transcription factor A and single-stranded DNA-binding protein. EMBO Rep 3, 451-456.Anti-TFAM home made
Takamatsu, C., Umeda, S., Ohsato, T., Ohno, T., Abe, Y., Fukuoh, A., Shinagawa, H., Hamasaki, N., and Kang, D. (2002). Regulation of mitochondrial D-loops by transcription factor A and single-stranded DNA-binding protein. EMBO Rep 3, 451-456.Anti-SDHA abcam CAT# ab14715
https://www.abcam.com/sdha-antibody-2e3gc12fb2ae2-ab14715.html
Anti-SDHB SANTA CRUZ CAT# sc-25851
https://www.scbt.com/p/sdhb-antibody-fl-280?productCanUrl=sdhb-antibody-fl-280&_requestid=2387382
Anti-COX1 abcam CAT# ab14705
https://www.abcam.com/mtco1-antibody-1d6e1a8-ab14705.html
Anti-COX2 abcam CAT# ab79393
https://www.abcam.com/mtco2-antibody-epr3314-ab79393.html
Anti-COX4 MBL CAT# PM063
https://ruo.mbl.co.jp/bio/e/dtl/A/?pcd=PM063
Anti-ATPa abcam CAT# ab14748
https://www.abcam.com/atp5a-antibody-15h4c4-mitochondrial-marker-ab14748.html
Anti-Cytrate synthase CST CAT# 14309S
https://www.cellsignal.jp/products/primary-antibodies/citrate-synthase-d7v8b-rabbit-mab/14309?site-search-type=Products&N=4294956287&Ntt=14309s&fromPage=plp&_requestid=2961995
Anti-Fumarase CST CAT# 4567S
https://www.cellsignal.jp/products/primary-antibodies/fumarase-d9c5-rabbit-mab/4567?site-search-type=Products&N=4294956287&Ntt=4567s&fromPage=plp&_requestid=2962032
Anti-MDH2 CST CAT# 11908S
https://www.cellsignal.jp/products/primary-antibodies/mdh2-d8q5s-rabbit-mab/11908?site-search-type=Products&N=4294956287&Ntt=11908s&fromPage=plp&_requestid=2962060
Anti-ACO2 abcam CAT# ab110312
https://www.abcam.com/hsp60-antibody-1d11bd8-mitochondrial-marker-ab110312.html
Anti-DLAT abcam CAT# ab110332
https://www.abcam.com/pyruvate-dehydrogenase-e2-antibody-15d3g9c11-ab110332.html
Anti-PDHE1A abcam CAT# ab168379
https://www.abcam.com/pyruvate-dehydrogenase-e1-alpha-subunit-antibody-epr11098-ab168379.html
Anti-PDHE1B abcam CAT# ab155996

https://www.abcam.com/pdhb-antibody-epr11097b-ab155996.html
Anti-CPS1 abcam CAT# ab45956
https://www.abcam.com/cps1-antibody-ab45956.html
Anti-HADHA abcam CAT# ab54477
https://www.abcam.com/hadha-antibody-ab54477.html
Anti-SOD2 CST CAT# 13194S
https://www.cellsignal.jp/products/primary-antibodies/sod2-d9v9c-rabbit-mab/13194?site-search-type=Products&N=4294956287&Ntt=13194s&fromPage=plp&_requestid=2962217
Anti-DDDK -tag mAB Magnetic Beads MBL CAT# M185-11
https://ruo.mbl.co.jp/bio/e/dtl/A/?pcd=M185-11
Anti-rabbit IgG, HRP-Linked GE CAT# NA934-1ML
https://www.sigmaaldrich.com/catalog/product/sigma/gena9341ml?lang=ja&region=JP
Anti-mouse IgG, HRP-Linked GE CAT# NA931-1ML
https://www.sigmaaldrich.com/catalog/product/sigma/gena9311ml?lang=ja&region=JP
TidyBlot™ Western Blot Detection Reagent:HRP Biorad CAT# STAR209P
https://www.bio-rad-antibodies.com/reagent/tidyblot-ac

Eukaryotic cell lines

Policy information about cell lines

Cell line source(s)	HeLa and HEK cells. The stable cell lines used in the study were derived from the corresponding parental cells.
Authentication	HeLa and HEK cells were purchased from ATCC and Thermo Fisher Scientific, respectively.<br>https://www.atcc.org/products/all/CCL-2.aspx<br>https://www.thermofisher.com/order/catalog/product/R78007#/R78007
Mycoplasma contamination	All cell lines tested were negative for mycoplasma comtamination.
Commonly misidentified lines<br>(See ICLAC register)	No commonly misidentified lines were used.
